# Nanomedicine in Ovarian Cancer: Advances in Imaging, Targeted Delivery, and Theranostic Therapeutic Platforms

**DOI:** 10.3390/cancers18010086

**Published:** 2025-12-27

**Authors:** Dorota Bartusik-Aebisher, Izabella Wilk, David Aebisher

**Affiliations:** 1 Department of Biochemistry and General Chemistry, Collegium Medicum, Faculty of Medicine, University of Rzeszów, 35-310 Rzeszów, Poland; dbartusikaebisher@ur.edu.pl; 2 English Division Science Club, Collegium Medicum, Faculty of Medicine, University of Rzeszów, 35-310 Rzeszów, Poland; iw135750@stud.ur.edu.pl; 3 Department of Photomedicine and Physical Chemistry, Collegium Medicum, Faculty of Medicine, University of Rzeszów, 35-310 Rzeszów, Poland

**Keywords:** ovarian cancer nanomedicine, targeted drug delivery, multimodal imaging, theranostic nanoparticles, intraperitoneal nanotherapy, tumour microenvironment (TME) targeting, stimuli-responsive nanocarriers, theranostic therapeutic platforms

## Abstract

Ovarian cancer continues to be the most lethal gynaecological malignancy, principally due to its late-stage diagnosis, extensive peritoneal dissemination, chemoresistance, and limitations of current imaging and therapeutic strategies. By optimising pharmacokinetics, refining tumour-selective drug delivery, and supporting high-resolution, multimodal imaging, nanomedicine offers a versatile platform to address these limitations. In this review, current progress across lipid-based, polymeric, inorganic, hybrid, and biomimetic nanocarriers is synthesised, emphasising how tailored physiochemical properties, surface functionalisation, and stimuli-responsive designs can improve tumour localisation, surmount stromal and ascetic barriers, and enable controlled drug release. Concurrently, significant advancement in imaging nanoprobes, including magnetic resonance imaging (MRI), positron emission tomography (PET)/single-photon emission computed tomography (SPECT), optical, near-infrared imaging (NIR), ultrasound, and photoacoustic systems, has evolved early lesion detection, intraoperative guidance, and quantitative monitoring of treatment. Diagnosis and therapy are further integrated within single platforms by emerging theranostic constructs, encouraging real-time visualisation of drug distribution and treatment response. Additionally, immune-nanomedicine, intraperitoneal depot systems, and nucleic acid-centred nanotherapies offer promising strategies to address immune suppression and molecular resistance in advanced ovarian cancer. In spite of noteworthy achievements, clinical translation is limited by complex manufacturing requirements, challenges with safety and stability, and restricted patient stratification. To unlock the full clinical potential of nanotechnology in ovarian cancer management, constant innovation in scalable design, regulatory standardisation, and integration of precision biomarkers will be necessary.

## Simple Summary

The major advances in nanomedicine for ovarian cancer are outlined in this review, underlining how engineered lipid, polymeric, inorganic, hybrid and biomimetic platforms improve imaging accuracy, intraperitoneal delivery, and therapeutic precision. Targeted ligands, stimuli-responsive release, and stealth coatings are key design principles which are demonstrated alongside applications in magnetic resonance imaging (MRI), positron emission tomography (PET)/single-photon emission computed tomography (SPECT), near-infrared imaging (NIR), ultrasound, and multimodal imaging. Nucleic acid nanotherapies, photothermal and enzyme-responsive systems, immune-nanomedicine and intraperitoneal depot strategies are also discussed in this article. The importance of standardisation and personalised nanotherapeutic approaches in ovarian cancer is highlighted by the evaluation of biodistribution, safety, manufacturing, and regulatory framework challenges.

## 1. Introduction

In 2020 ovarian cancer remained a high-mortality disease in Europe, with 39,000 new cases and 27,000 deaths reported, corresponding to an age-standardised incidence rate (ASR) of 15.5/100,000 and an ASR mortality of 10.3/100,000 [1]. Declining mortality trends with a predicted ASR of 4.32 per 100,000 women in 2022, as estimated by subsequent population-based analysis across Europe [2]. Despite these declines, updated estimates based on Global Cancer Observatory (GLOBOCAN) 2022 data show the ovarian cancer burden in Europe to continue to be substantial, with continually high incidence and mortality rates compared to other regions [3]. Ovarian cancer is a primary target for nanomedicine strategies in imaging, targeting and therapy, as these high mortality rates reflect late-stage symptom presentation, with most cases diagnosed at International Federation of Gynaecology and Obstetrics (FIGO) stage III or IV. Peritoneal metastatic spread, typical of HGSOC, and treatment resistance also contribute to this elevated mortality rate [4].

Biomarkers, such as CA-125 and human epididymis protein (HE4), showing low sensitivity and non-specific symptoms in the early stages of disease, remain the primary limitations of early detection of ovarian cancer, despite advances [5]. The window for effective intervention is reduced, as this delay often leads to diagnosis at advanced stages. Around 70% of patients presenting with epithelial ovarian carcinoma relapse following first-line therapy, presenting recurrence to be exceedingly common [6]. Therapeutic effectiveness is concurrently undermined by drug resistance, mediated by mechanisms such as DNA damage repair changes, altered transmembrane drug transport, epigenetic modifications and signalling pathway dysregulation [7]. These barriers pose direct challenges in the context of nanomedicine, as high-sensitivity targeted imaging and biomarker-loaded nanoprobes are demanded for early detection, while resistance and recurrence require nanocarriers that overcome residual disease niches and heterogeneity and are capable of overcoming efflux pumps. Crucial intervention points for imaging, targeting and therapeutic nanomedicine in ovarian cancer are highlighted by the triad of frequent relapse, chemoresistance and late detection [8].

Cytoreductive surgery and platinum-taxane chemotherapy are relied on as the standard treatments of advanced ovarian cancer; although, microscopic peritoneal implants often fail to be completely removed during surgery, and residual disease remains an utmost source of relapse [9]. The development of drug resistance mechanisms, for example, enhanced DNA repair or efflux pumps, in tumour cells, dose-limiting toxicities and non-specific biodistribution hinder systemic chemotherapy [10]. Nanoparticle-based platforms aim to utilise the therapeutic window for nanomedicine by combating these limitations. These platforms intend to penetrate residual microscopic disease, unreachable by standard chemo or surgery, improve tumour-specific delivery, and decrease off-target toxicity [11]. Challenges such as tumour heterogeneity, abnormal peritoneal fluid dynamics, and integration into current surgical/chemotherapeutic workflows, nonetheless, need to be overcome for effective translation of nanomedicine.

The improvement of pharmacokinetics (PK), enabling targeted delivery, and adding real-time imaging are solutions offered by nanomedicine to overcome ovarian cancer’s therapeutic impediments. Reduced cardiotoxicity and sustained activity in relapsed disease can be achieved by pegylated liposomal encapsulation, exemplifying PK gains. Liposomal encapsulation: shrinks volume of distribution, boosts tumour exposure versus free drug and extends circulation half-life [12]. Although recognised limitations of pegylated liposomes, such as the accelerated blood clearance (ABC) phenomenon in particular, counterbalance these benefits following repeated dosing. Additionally, rapid opsonisation and hepatic uptake, which can lead to reduced circulation time, reduced tumour accumulation, and unpredictable therapeutic performance, are promoted by the generation of anti-PEG IgM antibodies. Non-specific biodistribution and dose-limiting toxicity, inherent to conventional chemotherapy, can aim to be overcome by targeting moieties (e.g., peptides, antibodies, and folate derivatives) attached onto nanocarriers, which will concentrate the active compound at ovarian cancer cells or within their microenvironment [13], while imaging-capable nanoparticles allow earlier detection and surgical guidance, beyond delivery. Further, contrast-enhanced platforms for MRI, PET/SPECT, and optical/near-infrared (NIR) modalities, including intraoperative probes designed for peritoneal micrometastases, are catalogued in ovarian cancer-focused reviews [14]. Intra-abdominal tumour visualisation during cytoreductive surgery can be illustrated with preclinical work with erythrocyte-derived NIR nano-constructs in support of the theragnostic concept “see and treat” [15]. Together these advances address three needs: Drugs are kept in circulation and within tumours longer, optimising PK; intratumoral drug-to-healthy-tissue ratios are increased by molecular targeting; and disease is mapped, resection is guided, and response is monitored by longitudinal or real-time imaging. Although clinical translation is still hindered by heterogeneity and peritoneal transport barriers [16], nanomedicine is positioned as a practical complement and, in certain scenarios, an enhancer to systemic chemotherapy and surgery due to the maturation of ovarian-specific nanoplatforms across delivery and diagnostic fronts.

## 2. Biological and Pathophysiological Context for Nanomedicine Delivery

### 2.1. Tumour Microenvironment, Peritoneal Dissemination and Ascites

**Figure 1:**
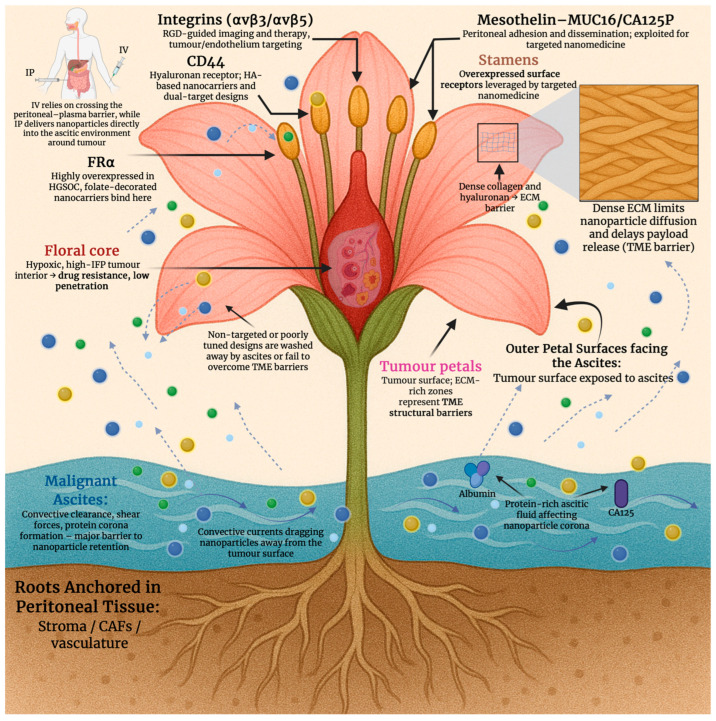
Conceptual “tumour flower” model illustrating the multilevel biological barriers that impede nanoparticle delivery in high-grade serous ovarian cancer (HGSOC), including ascites-driven clearance, ECM-rich tumour surfaces, intratumoural hypoxia, and overexpressed molecular receptors exploitable for targeted nanomedicine. Created in BioRender. Wilk, I. (2025) https://BioRender.com/l239u55.

Formidable physical and biochemical barriers to nanomedicine delivery are imposed by the tumour microenvironment (TME) in ovarian cancer. Aberrant, leaky vasculature and inadequate perfusion cause regions of hypoxia that promote drug resistance and limit oxygen-dependent imaging probes by triggering hypoxia-inducible factor 1, alpha subunit (HIF-1a)-mediated changes [17]. Compressed lymphatics and abnormal vessel outflow elevate interstitial fluid pressure (IFP); the extravasation of nanoparticles into the tumour interstitium is further impeded by this, and rather than retention, convective clearance is favoured [18]. Dense stromal architecture, which restricts nanoparticle diffusion and delays payload release, is a consequence of extensive extracellular matrix (ECM) remodelling, as seen in Figure 1. This includes increased collagen deposition, hyaluronic acid accumulation and activation of cancer-associated fibroblasts [19]. Hypoxia, high IFP, and dense ECM must be addressed by nanomedicine design in ovarian cancer. This can be performed by enhancing tumour penetration, imaging contrast, and therapeutic efficiency through the integration of stimuli-responsive release, optimised carrier architecture, and stromal targeting features.

Effective nanomedicine delivery in advanced ovarian cancer is significantly obstructed by peritoneal dissemination and accumulation of malignant ascites. Nanoparticle residence time is reduced, and diffusion into implants is limited by convective clearance generated by tumour nodules bathed in ascitic fluid and seeded on peritoneal surfaces [20]. Nanoparticle attachment and retention on the peritoneal surface is impeded by the fluid-driven shear and movement of ascites. Furthermore, due to hepatic clearance and limited transport across serosal membranes, systemic intravenous delivery faces poor peritoneal penetration [21]. Thus, local concentration can be enhanced by the emergence of intraperitoneal administration of nanoparticles as a strategic route. Rapid drainage and nanoparticle clearance in ascitic fluid, even in this route, require design features such as prolonged retention, large tumoral surface binding, and size optimisation to improve uptake and therapeutic index in disseminated peritoneal disease [22].

### 2.2. Molecular Targets

Anchors for targeted nanomedicine in EOC are provided by several cell surface biomarkers that are frequently overexpressed. In high-grade serous disease, FOLR1 (FR-α) is enriched, and folate-directed therapeutics and diagnostics are underpinned by it. Subtype distribution is quantified, and assay standardisation needs are highlighted in recent clinic-pathologic studies [23]. While mesothelial adhesion and peritoneal dissemination are mediated by mesothelin (MSLN) binding MUC16/CA125, this is an axis widely leveraged for nanoparticle and antibody/nanobody targeting [24]. Further, epithelial tumour cells are marked by epithelial cell adhesion molecule (EpCAM), and it has been linked to prognosis and chemoresistance. EpCAM is used for EV-based readouts and surface functionalisation with targeting ligands or aptamers [25]. The hyaluronan receptor, CD44, supports hyaluronic acid (HA)-based carriers or dual-target designs, whilst enriching stem-like subpopulations [26]. Whereas RGD-guided imaging/therapy and intraperitoneal targeting in ovarian models are enabled by integrins (avβ3/avβ5) on tumours and antagonistic endothelium [27], as shown in Figure 1. Although its prognostic value is heterogeneous, it is important to mention PD-L1 as a marker variably expressed in EOC. It plays a significant role in immuno-nanomedicine combinations and immune-responsive theranostics [28]. Ligand selection, patient stratification, and payload placement are informed by these biomarkers for imaging, targeting, and therapeutic nanoplatforms in ovarian cancer. Table 1 summarises the key molecular targets seen in ovarian cancer.

### 2.3. Routes of Administration

**Figure 2:**
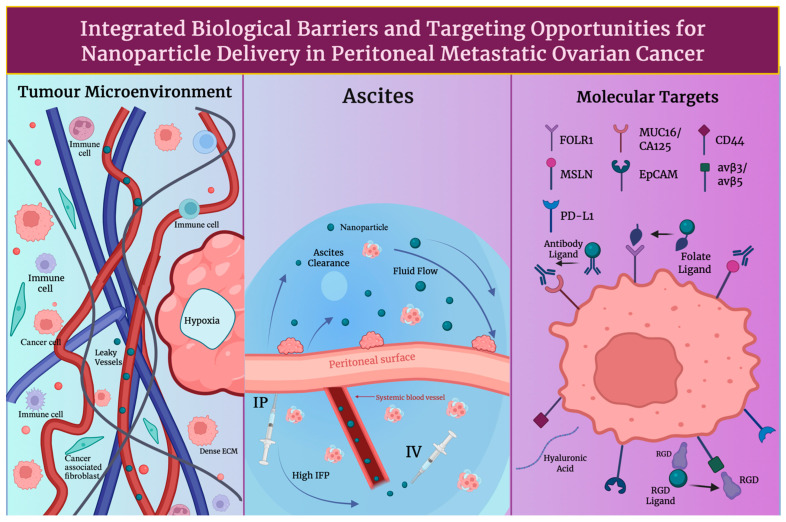
Key tumour and peritoneal barriers affecting nanoparticle transport after intravenous (IV) versus intraperitoneal (IP)delivery. Shown are TME features, ascites fluid dynamics, and surface receptors that determine peritoneal exposure, clearance, and tumour uptake. Created in BioRender. Wilk, I. (2025) https://BioRender.com/l239u55.

The exposure of peritoneal implants is crucially shaped by route selection. The standard route remains as intravenous (IV) therapy, although drug transfer to surface-embedded nodules is limited by the peritoneal plasma barrier and systemic clearance [37], as seen in Figure 2. After IV administration, typically only 0.1–2% of the injected nanomaterial dose accumulates in solid tumours, with noticeable inter-lesional variability. At the peritoneal surface, higher locoregional concentrations can be achieved by intraperitoneal (IP) delivery as a route of administration. IP delivery has also shown survival advantages in meta-analysis and randomised trials [38]. Patient selection and supportive care are informed by common challenges, such as toxicity and abdominal discomfort, as shown by patient-reported outcomes from GOG-172 [39]. However, hyperthermic intraperitoneal chemotherapy (HIPEC) is preferred in a surgical environment; following interval cytoreduction, HIPEC is shown to increase recurrence-free and overall survival in randomised trials. Data from long-term analysis also reinforces its strengths [40]. In primary and certain recurrent diseases, a 2022 meta-analysis also shows HIPEC to be of benefit to patients, while heterogeneity is mentioned in indications and regimens [41]. Long-circulating, stealth carriers with active targeting to reach peritoneal deposits are favoured for IV administration in nanomedicine. Contrastingly, designs that resist ascitic clearance and have the ability to remain stable in mild hyperthermia (41–43 degrees Celsius) are valued in IP/HIPEC [42]. Thus, peritoneal exposure and therapeutic index in ovarian cancer can be improved by prolonged half-life and tumour homing in IV administrative routes and increasing peritoneal surface adhesion, nanoparticle diameter optimisation and thermal stability in IP/HIPEC routes [43].

### 2.4. Clinical Relevance and Limitations of the EPR Effect in Humans

The enhanced permeability and retention (EPR) effect in humans is real but heterogenous: therefore, rather than being a reliable default, its effectiveness depends on patient-specific and lesion-specific biology, which includes vascular permeability, perfusion and stromal composition. Tumour uptake of nanomedicines has been confirmed by clinical imaging studies, but significant inter- and intra-individual variability is consistently observed [44]. In patients, a 35-fold difference in liposome deposition across metastatic lesions, together with an association between higher deposition and more favourable outcomes, was quantified by PET/CT with ^64^Cu-MM-302 (HER2-targeted pegylated liposomal doxorubicin), which supported the concept of image-guided patient selection [45]. The extent of this variability is particularly relevant in ovarian cancer because the disease often presents with disseminated peritoneal implants showing heterogenous vascularisation and perfusion. Consequently, in poorly perfused lesions, EPR-only platforms may underperform, motivating the integration of active targeting, tumour microenvironment modulation, and theranostic data to personalise delivery strategies [46]. In this regard, nanomedicine strategies in ovarian cancer increasingly pair quantitative imaging modalities (PET/SPECT/MRI) with therapeutic payloads to map nanoparticle deposition, adjust dosing, and combine passive EPR with molecular ligands, such as folate or CD44, or stimuli-responsive release mechanisms [47], and in doing so, overall theranostic performance is significantly improved.

## 3. Nanocarrier Platforms and Engineering Design

In the context of ovarian cancer, nanocarrier engineering increasingly follows a translational logic in which design choices are evaluated for imaging compatibility, patient stratification, and clinical feasibility, in addition to delivery performance. Several recurring design principles, including particle size control, surface chemistry and stealthing, stimulus-responsive release, and targeting strategy, govern performance in ovarian cancer. While material composition dictates imaging or therapeutic modality compatibility, how these shared parameters are tuned to the peritoneal tumour microenvironment primarily determines translational success.

### 3.1. Lipid-Based Systems

Polyethylene glycolated (PEGylated) liposomes increase circulation time, decrease cardiotoxicity, and are demonstrated in recurrent ovarian cancer. Pegylated liposomal doxorubicin (PLD), Doxil and Caelyx, showed improved results in platinum-sensitive disease, whilst exceeding topotecan’s effectiveness; PLD carboplatin is also considered an efficient treatment [48]. Intravascular, hyperthermia-induced rapid release with image-guided dosing is enabled by thermosensitive liposomes; lessons acquired from translational research and empirical design guidelines for thermosensitive liposome development are provided in a well-established body of research [49]. Nanocarriers are often manipulated with surface ligands to recognise and bind to molecular biomarkers; for example, folate ligands/antibodies are used to bind to folate receptor α (FAα), a characteristic ovarian cancer molecular marker. Peptides, such as arginine-glycine-aspartic acid (RGD) or aptamers, can be used to increase absorption and facilitate biomarker-guided patient selection [50]. Furthermore, biodistribution and response can be monitored during imaging by PET or NIR probes being co-loaded onto liposomes or lipid nanoparticles (LNPs) [51]. To intercede endosomal escape, ionisable lipids, helper phospholipids, cholesterol, and PEG-lipids are used in LPNs for siRNA and mRNA. Whereas, in ovarian models, dioleoylphosphatidylcholine (DOPC)-based formulation, known as EPHARNA, successfully silenced the EphA2 gene and achieved antitumour activity; here local drug exposure can be maximised by IP administration [52]. Nanocarrier diameter, PEG surface density, TME activators (pH/redox) and administration route have been shown to marginally progress transport across ascites-rich peritoneal environments and to regulate the very variable EPR effect in ovarian cancer models. Although, it is important to mention that these strategies do not uniformly overcome ascites-associated transport barriers, and rather than decreasing EPR, EPR-mediated accumulation aims to be exploited by contemporary nanocarrier designs, where present, while integrating complementary mechanisms, such as intraperitoneal delivery, active targeting, or stimuli-responsive release, in order to account for its heterogenicity and limited reliability in advanced disease [53].

### 3.2. Polymeric Nanoparticles

Adjustable size, surface chemistry, and stimuli sensitivity for systemic and intraperitoneal delivery are enabled by polymeric nanocarriers, making them fundamental to ovarian cancer nanomedicine. Polylactic-co-glycollic acid (PLGA) nanoparticles provide biodegradability and substantial drug/siRNA co-loading; surface functionalisation, for example, folate or hyaluronan for CD44; as well as peritoneal tumour retention, endosomal escape, and reversion of chemoresistance, for example, HA-PLGA nanoparticles co-loaded with paclitaxel (PTX) and/or small interfering RNA targeting focal adhesion kinase (FAK siRNA) [54,55]. Hydrophobics, such as PTX, can be solubilised by PEG-poly lactic acid (PLA) copolymer micelles. These micelles can also enable dose increase, and they can be modified for pH-induced PEG detachment and endosomal escape. Due to PEG-PLA micellar formulations being clinically established and manufacturable, their translation to ovarian cancer applications is well supported [56]. Poly-amidoamine (PAMAM) is an example of a dendrimer that can offer multivalent, well-defined structures for siRNA/miRNA delivery against resistance mediators, such as twist-related protein 1 (TWIST) and p70S6K. This can be used in combination with PTX to improve antitumour efficiency by being made redox/reactive oxygen species (ROS) reactive for TME activation [57]. Cathepsin or pH-cleavable linkers are used by polymer drug conjugates (PDCs) and antibody polymer drug conjugates for controlled release and can multiplex drugs, for example, gemcitabine and doxorubicin, or they can be used to add immune targeting, such as programmed death ligand 1 (PD-L1). This can lengthen survival in ovarian models and, when paired with imaging labels, supports theranostics [58]. Ascites and peritoneal nodule penetration are improved across platforms targeting moieties, such as FAα, IP delivery and tumour-penetrating peptides. Whilst platinum and taxane resistance is addressed by co-delivery of chemotherapeutic agents with RNA interference (RNAi) or poly (ADP-ribose) polymerase (PARP)/70-kDa ribosomal protein S6 kinase 1 (P70S6K) inhibitors. Design confluence toward compact, stable, and activatable nanocarriers, illustrated in recent PLGA-PTX hybrids and mixed micelles, shows optimisation for ovarian cancer imaging-drug delivery workflows [59].

pH- and redox-responsive release mechanisms are frequently integrated across lipid and polymeric nanocarriers, enabling spatially controlled payload activation in ovarian cancer models, as discussed in Section 3.5.

### 3.3. Inorganic and Hybrid Systems

Multimodal imaging, targeting, and therapy are allowed by inorganic and hybrid nanoplatforms in ovarian cancer. Simple surface functionalisation for antibody or aptamer targeting is offered by gold nanoparticles, including spheres, rods and shells. Additionally, these nanoparticles provide powerful plasmonics for photoacoustic (PA) or photothermal therapy and drug conjugation; the latest ovarian-focused reviews emphasise progress towards precision oncology and overcoming clinical obstacles [60]. Mesoporous silica nanoparticles (MSNs) supply high capacity and adjustable pores. ‘Wormhole’ MSNs have been successful in achieving TME-targeted theranostics and optoacoustic discovery of ovarian lesions. Further, radioisotope-loaded holmium-166-loaded mesoporous silica nanoparticles (166Ho-MSNs) can show peritoneal metastasis control with restricted off-target dose [61], whereas MRI contrast and magnetic hyperthermia are enabled by iron oxide nanoparticles (IONPs). Heating efficiency can be improved by engineering multicore IONPs and biocompatible nanoclusters, whilst deep stimulation for apoptosis and vascular disturbance can be permitted by alternating magnetic fields [62]. Quantum dots (QDs) can provide a bright and stable fluorescence, useful for intraoperative guidance and selected drug-aptamer conjugates, such as mucin 1, cell surface associated (MUC1) aptamer-doxorubicin, that pair chemotherapy with imaging in ovarian models [63,64]. Lastly, magnetic MSNs, or plasmonic silica shells, are examples of hybrid core-shell designs that can multiplex functions such as loading cytotoxins or siRNA, adding stimuli-sensitive release, and combining PA/MR/fluorescence signals. This supports image-guided therapy and ascites/peritoneal nodule management [65]. The use of antifouling coatings to navigate peritoneal fluid, radiotheranostic agents for intraperitoneal disease, and clinically orientated manufacturing remain as ongoing priorities [66].

### 3.4. Surface Functionalisation and Stealthing

Suppression of opsonisation, prolonging of circulation time, and improvement of drug delivery to tumours can be achieved by hydrophilic ‘stealth’ coatings in ovarian cancer nanocarrier design. The reason behind this hydrophilic requirement is because a steric and energetic barrier that reduces nonspecific protein absorption and protein corona formation develops when highly hydrated polymer layers bind water molecules. Hydrophilic coatings decrease rapid hepatic and splenic clearance by limiting opsonin binding and subsequent recognition by the mononuclear phagocyte system, and in doing so prolong circulation time and enhance tumour accumulation. PEGylation is the most reliable tool, demonstrating a reduction in protein adsorption and renal clearance. Although the ‘accelerated blood clearance’ effect, as well as anti-PEG antibodies, encourages other options [67]. Carboxybetaines and sulfobetaines are examples of zwitterionic polymers that show elongated circulation, decreased complement stimulation, and sensitive designs appropriate for drugs and genes. Zwitterionic nanoparticles, such as upper critical solution temperature (UCST) type and hyperthermia-activated systems, are used in ovarian cancer models to improve paclitaxel efficiency and retention [68]. In addition, wrapping nanocarrier cores with membranes from platelets, RBCs, leukocytes or sometimes even ovarian cancer cells is called biomimetic cloaking. This process facilitates immune evasion, homotypic binding, and multivalent targeting, ideal for imaging and therapy, by adding ‘self’ markers, such as cluster of differentiation 47 (CD47) and adhesion ligands [69].

Stealthed nanocarriers in combination with epithelial or stromal targets, such as FRα, tumour endothelial marker 1 (TEM1) and luteinising hormone releasing hormone (LHRH), in addition to layer-by-layer polymers, to achieve ‘target and release’, radiation sensitisation and multi-imaging modalities, such as photoacoustics or CT, while alleviating peritoneal clearance, are being used for ovarian cancer [70]. Ovarian cancer nanomedicine reviews emphasise PEG, zwitterion, and membrane-coated platforms that can co-deliver siRNA or chemo, aid image-guided interventions, and strengthen immunotherapy. Key design parameters involve graft density, membrane isolation purity, valency of ligand, and deterioration to balance stealth with tumour penetration and endosomal escape [71].

### 3.5. Triggered and Controlled Release Mechanisms

Triggered or controlled release designs use tumour-specific cues to achieve site-specific drug release in ovarian cancer. pH, redox and enzymes are considered endogenous triggers. In pH-triggered release designs, acidic TME or endosomes can dissolve acid-labile linkers, or the swelling of ionisable matrices can accelerate release and endosomal escape. Intratumoral deposition and efficiency can be improved by multiple pH-sensitive polymer/lipid systems [72]. Moreover, redox-controlled release designs rely on increased glutathione (GSH) or reactive oxygen species (ROS) to cleave disulphide or thioketal linkers, allowing for on-the-spot drug or siRNA release and combination therapies [73]. Whereas, in enzyme-triggered release designs, cathepsins or matrix metalloproteinases (MMPs) in peritoneal metastases degrade shells or cut peptide linkers during selective unloading [74]. Protein corona-resistant surfaces, such as zwitterionic or antifouling, preserve targeting and release kinetics by maintaining trigger accessibility in ascitic fluid [75].

Stimuli-responsive control is provided by exogenous triggers. In designs that are heat responsive, heat-based treatment is coupled with controlled drug delivery. Heat-based treatment mechanisms extend beyond conventional hyperthermia and include magnetic hyperthermia using superparamagnetic iron oxide nanoparticles (SPIONs), which are exposed to alternating magnetic fields (AMFs); photothermal therapy via NIR-absorbing nanomaterials; and thermosensitive carriers that release payloads above set transition temperatures. Spatially controlled cytotoxicity, heat-enhanced drug diffusion, and synergistic chemo- or phototherapy, particularly relevant for intraperitoneal disease, are enabled by these heat-based strategies. Magnetic field hyperthermia (MFH)-enabled chemotherapy with MRI monitoring in ovarian cancer models [76] and NIR-activated gold nanostructures, which enable spatially precise photothermal ablation or drug release [77], are included in representative examples. Further, carriers that rely on magnetic fields can concentrate drugs and, when combined with AMF, can combine heat-induced release and targeting [78]. Free paclitaxel in ovarian cancer cells is being outperformed by developing UCST zwitterionic nanogels, which can trigger drug release under mild hyperthermia [79].

**Figure 3:**
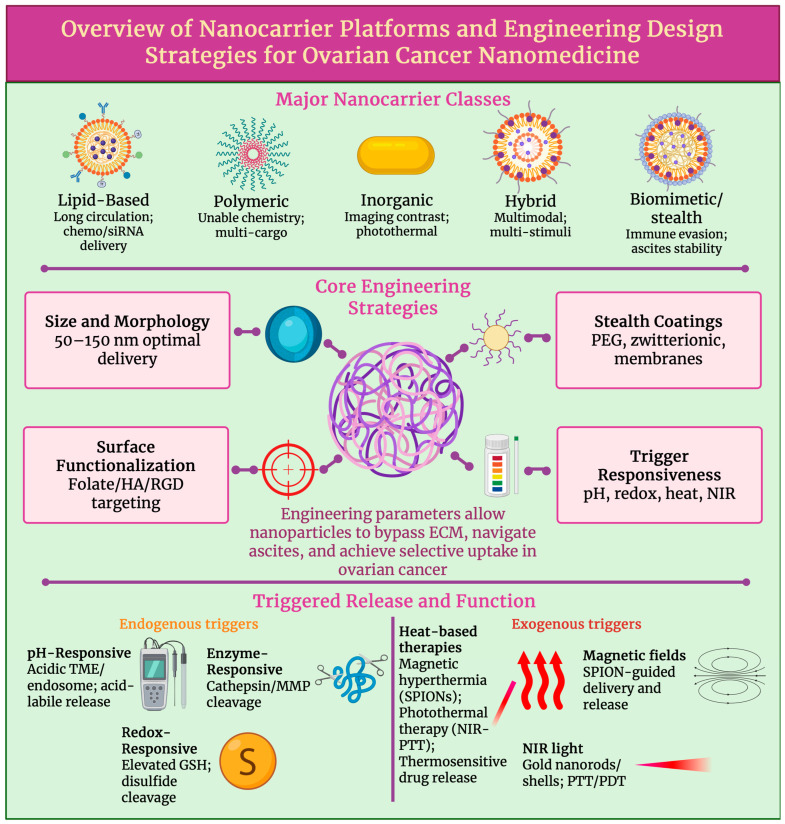
Integrated framework of nanocarrier platform types, engineering design parameters, and stimuli-responsive mechanisms relevant to ovarian cancer nanomedicine, highlighting design and trigger principles. Created in BioRender. Wilk, I. (2025) https://BioRender.com/r1lyg2v.

We can see that precise and on-demand drug release can be achieved by multi-stimuli strategies: by pH, redox and enzymatic (endogenous) and thermal, photonic and magnetic (exogenous) triggers, as shown in Figure 3. This can advance peritoneal retention, response and tumour penetration, while minimising off-target toxicity, which is essential for image-guided, combination treatment in ovarian cancer [80]. Table 2 summarises the key nanocarrier platforms and engineering designs in ovarian cancer nanomedicine.

### 3.6. Design Scalability and Clinical Translation

Design scalability and clinical translation of ovarian cancer nanocarriers rely on production-ready formulations, such as PLGA and liposomes with modifiable surfaces, size and drug release, while maintaining batch-to-batch reproducibility and pharmacokinetics. In a clinical example, solid tumours were treated with albumin-bound paclitaxel nanoparticles, known as Nab-paclitaxel; this demonstrated that consistent pharmacokinetics and enhanced safety can be achieved by a scalable production process in which nanoparticle size was controlled and albumin coating and reproducible drug loading were involved, therefore acting as a standard for clinical translatability in nanomedicine [88]. Rigorous analytical profiling, for example of hydrodynamic size, polydispersity index (PDI) and drug-to-lipid ratio, correlated with critical quality attributes under the International Council for Harmonisation (ICH) guidelines, specifically Q8, quality by design (QbD), supports regulatory filings and reduces variability [89]. Additionally, standardisation of analytical methods and aligning with developing nanomedicine standards strengthens reproducibility [90]. Opsonisation and off-target toxicity are decreased by biocompatible engineering, such as PEGylation and biodegradable backbones, like PLGA, while allowing tumour localisation in ovarian models [91]. Translation is frequently limited by storage stability; particle integrity and shelf life are preserved by controlled reconstitution and lyophilisation with optimised cryoprotectants. Whereas frozen or lyophilised formats can preserve encapsulation and activity of lipid nanoparticles [92]. Process intensification, which can be performed by inline mixing and the use of closed systems, as well as by authenticated scale-up models connecting minor-scale clinical process parameters (CPPs) to manufacturing-scale batches for scalability, addresses batch-to-batch variation across sites, commonly highlighted in preclinical-to-clinical evaluations [93]. Lastly, regulatory alignment now benefits from clearer guidance globally that is specific to products which are nano-enabled, and complex injectables are more frequently used by regulatory agencies. These are trends which can be seen in recent surveys and overviews in the industry, but they also demand enhanced stability and immunogenicity [94].

Together, these engineering parameters not only control nanocarrier stability and delivery efficiency but also determine compatibility with imaging data, patient stratification strategies, and subsequent clinical translation.

## 4. Targeting Strategies in Ovarian Cancer Nanomedicine

### 4.1. Active and TME Targeting Strategies in Ovarian Cancer Nanomedicine

Active targeting in ovarian cancer nanomedicine often makes use of antigens that are evidently overexpressed on tumourous tissue, in comparison to their limited distribution in healthy tissue. On many high-grade serous tumours, folate receptor-α (FRα) is expressed in abundance. FRα has been used to design theranostic nanoemulsions and micelles that can enable MRI monitoring whilst concentrating platinum drugs in peritoneal metastases [95]. When antibody-loaded paclitaxel nanoparticles and anti-MUC16 nanomicelles have been used to target mesothelin and MUC16 (CA125), ovarian cancer targeting ability and tumour uptake were increased, and systemic toxicity was reduced [96,97]. Additionally, non-invasive MRI and optical imaging, with tumour-specific accumulation in human epidermal growth factor receptor 2 (HER2)-positive xenografts, is facilitated by HER2-targeted iron oxide nanoparticles tagged with cisplatin [98]. This concept is extended to circulating or minimal residual disease by EpCAM-directed magnetic particles, which are described in the molecular imaging and contrast agent database (MICAD), as well as by other related imaging probes [99]. Further, siRNA or photosensitisers are delivered to avß3-positive ovarian tumour and endothelial cells by integrin-targeted RGD-labelled nanoparticles, which enable the concurrent attack of tumour cells and angiogenic vasculature [100]. Hyaluronic acid-decorated carriers frequently exploit CD44 to deliver cytotoxins [101] or to deliver MDR1 siRNA selectively to chemoresistant CD44-overexpressing ovarian cancer cells [102]. In addition, complementary TME targeting focuses on tumour-associated macrophages (TAMs), fibroblasts and vasculature. Following accumulation in ovarian TAMs, mRNA or manganese-loaded nanocarriers and large anionic nanoparticles can reprogramme TAMs towards antitumour phenotypes or increase the effectiveness of chemotherapy [103]. On activated macrophages, folate receptor ß is selectively expressed and has been widely used in folate-targeted macrophage nanotherapies, therefore offering a selective entry point for TAM-directed nanomedicine in ovarian cancer [104]. Co-targeting of tumour cells and fibroblasts, remodelling of extracellular matrix and enhancement of immune infiltration in ID8 ovarian models can be achieved by cancer-associated fibroblast mimetic or cancer-associated fibroblast (CAF) hybrid membrane-coated nanoparticles [105]. A route to inhibit angiogenesis is provided by HA-coated siRNA nanocarriers targeting CD44 on tumour endothelium [106]; this integrates vascular, stromal and immune targeting into multifunctional ovarian cancer nanomedicines.

### 4.2. Overcoming Receptor Heterogeneity Through Dual-Targeted and Theranostic Nanoparticle Platforms

**Figure 4:**
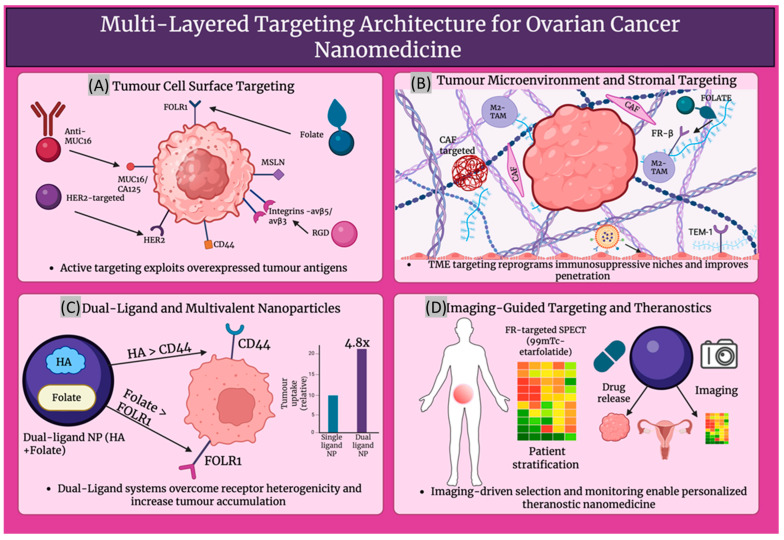
Describes; (**A**) Targeting tumour surface receptors. (**B**) Modulating the tumour microenvironment. (**C**) Dual-ligand nanoparticles improving uptake. (**D**) Imaging-guided targeting enabling personalised theranostics. Created in BioRender. Wilk, I. (2025) https://BioRender.com/vuk44v2.

The marked receptor heterogenicity of ovarian cancer is being addressed by increasingly exploited multivalent and dual ligand nanoparticle systems. A significantly higher uptake in SKOV3 ovarian cancer cells and a 4.82-fold higher tumour accumulation in vivo than single ligand control, as shown in Figure 4, was exhibited by dual CD44 and folate receptor-targeted hyaluronic acid-ceramide folic acid nanoparticles, showing synergistic targeting and improved visualisation of intraperitoneal tumours [107]. More broadly, it has been established by dual-ligand gold nanoparticles that multivalent engagement of two receptors can considerably improve cell recognition and differentiation between high- and low-expressing tumour cells, providing conceptual support for dual-receptor nanomedicine in heterogenous ovarian tumours [108]. Targeting is further integrated with imaging data to support image-guided treatment by theranostic platforms. Dual fluorescence and MRI contrast are provided by HER2-targeted gold nanoshell complexes, while NIR photothermal ablation of HER2-overexpressing, drug-resistant OVCAR3 ovarian cancer cells is enabled in vitro [109]. Biomarker-guided companion diagnostics have shown clinical translatability in folate receptor-positive, platinum-resistant ovarian cancer, where patients who are most likely to benefit from the FR-targeted drug conjugate vintafolide are selected using the FR-targeted SPECT tracer ^99m^Tc-etarfolatide [110], demonstrating imaging-based enrichment of nanotherapeutic trials. From a broader molecular profiling viewpoint, the key determinants for guiding personalised, nanoparticle-enabled therapy in ovarian cancer are highlighted as multi-omic profiling of BRCA/HRD, HER2, FOLR1 and other targets [111]. On this basis, Garbuzenko et al. suggest a bank of pre-synthesised, ligand-decorated nanocarriers whose formulation, either containing drugs, siRNAs or targeted peptides, is chosen ex vivo, in accordance with the patient’s tumour gene-protein signature, offering a practical framework for truly personalised ovarian cancer nanomedicine [112]. Table 3 describes the major receptor-ligand axes exploited in ovarian cancer nanomedicine.

## 5. Imagining and Theranostic Nanomedicine

Imaging nanomedicine in ovarian cancer follows a common theranostic logic across MRI, PET/SPECT, optical, ultrasound, and photoacoustic modalities: nanoparticles are engineered to accumulate at disseminated peritoneal lesions, biodistribution is reported quantitatively or intraoperatively, and where possible triggered therapy is enabled. Therefore, differences between modalities reflect signal physics instead of fundamentally distinct delivery strategies.

### 5.1. Magnetic Resonance Imaging (MRI)

High-resolution visualisation of ovarian cancer cells within the peritoneal cavity can be enabled by MRI nanoprobes. Strong T_2_ contrast is provided by superparamagnetic iron-oxide nanoparticles (SPIONs), and they can be used for ovarian tumour uptake after engineering. Folate-decorated Fe_3_O_4_ SPIONs generated marked negative contrast in tumours visualised within orthotopic intraperitoneal models. This supported theranostic use by enabling detection of peritoneal implants in preclinical models [125]. In addition to passive contrast, delivery and response by MRI has been simultaneously monitored by theranostic magnetic iron oxide platforms, enabling the detection of differences in accumulation of nanoparticles across heterogenous ovarian lesions and decreased peritoneal metastases in preclinical models. These platforms have also been used to deliver cisplatin to ovarian tumours [98]. Gadolinium (Gd)-labelled liposomes go beyond intravascular residence and intensify reflexivity for T1-weighted, positive-contrast imaging. Vigorous MR signal enhancement, which improved the visualisation of intraperitoneal tumours and complementary NIR signal, was achieved by dual-Gd liposomes co-loaded with indocyanine green (ICG). This enabled multimodal mapping of ovarian cancer, improving detection, treatment monitoring and surgical guidance [126]. Superior tumour MR contrast and adaptability to antibody or peptide functionalisation for ovarian applications have long been demonstrated by Gd-liposomal formulations, often using polychelating amphiphilic polymers, to raise Gd content [127]. Together, SPIONs and Gd-liposomes form the basis of MRI-based mapping of peritoneal metastases and facilitate theranostic workflows that unite lesion localisation with simultaneous delivery and on-treatment monitoring of ovarian cancer.

### 5.2. Nuclear Imaging (PET/SPECT)

Quantitative, whole-body tracking of the nanoparticle in ovarian cancer is facilitated by radiolabelled nanocarriers for PET and SPECT; this can provide continuous insight into tumour accumulation and circulation and can help spot off-target distribution. Characterisation of pharmacokinetics and optimisation of delivery strategies in nanomedicine have been progressed using liposomes and polymeric nanoparticles tagged with radionuclides such as ^99m^Tc, ^64^Cu or ^89^Zr [51]. Radiolabelled expansile nanoparticles exhibited high peritoneal tumour uptake in intraperitoneal tumour models, imitating advanced ovarian cancer. These nanoparticles approached 30% of the administered dose, and clear distinctions between intravenous and intraperitoneal delivery were revealed. This study highlighted the importance of nuclear imaging for treatment optimisation and biodistribution mapping [128]. Also emerging as theranostic agents are radiolabelled biologics and nanoparticles. PET-centred imaging of ovarian cancer lesions is permitted by ^99^Zr conjugated to anti-Müllerian-inhibiting substance type II receptor (MISRII) antibodies, while combined radionuclide treatment, which enables patient selection and customised dosimetry, is allowed by therapeutic analogues labelled with ^177^Lu or ^213^Bi [129,130]. Radiolabelled nanocarriers are described to function as non-invasive tracers in recent reviews, which state they can display therapeutic nanoparticle biodistribution and monitor response to treatment over time. Therefore, radiolabelled nanocarriers can play a significant role in precision management of ovarian peritoneal metastases [131], which drive most ovarian cancer-related mortality.

### 5.3. Optical and Near-Infrared (NIR) Imaging

**Figure 5:**
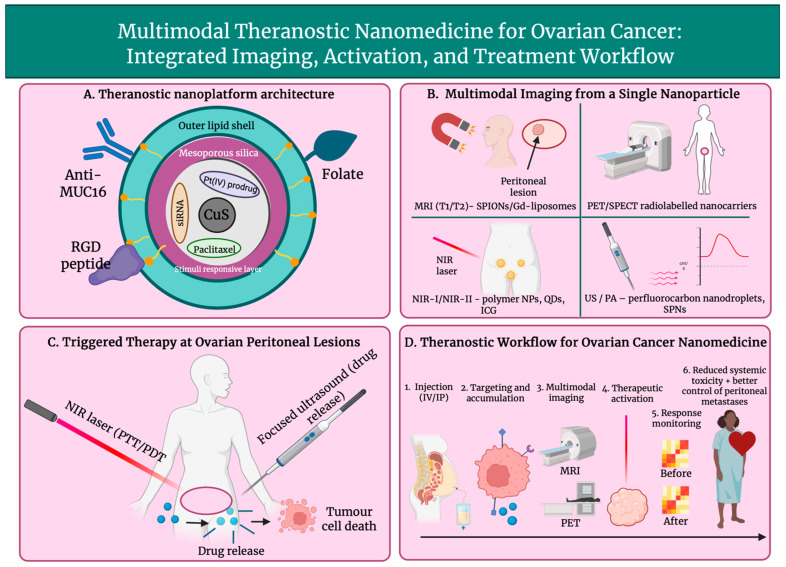
Overview of a multifunctional theranostic nanoparticle for ovarian cancer, showing nanoparticle architecture (**A**), multimodal imaging capabilities including MRI, PET/SPECT, NIR, and US/PA (**B**), triggered photothermal, photodynamic, and ultrasound-induced therapy at peritoneal lesions (**C**), and the integrated workflow from administration to response monitoring and improved disease control (**D**). Created in BioRender. Wilk, I. (2025) https://BioRender.com/vfi2pt8.

Intraoperative and metastatic localisation in ovarian cancer is increasingly relying on optical and NIR imaging, using NIR-fluorophores and QD nanocarriers. Improved tissue penetration, lowered autofluorescence, and enhanced tumour-to-background contrast can be provided by fluorophores producing emission in the first (NIR-I, 700–900 nm) or the second window (NIR-II, 1000–1700 nm). These features are crucial in enabling the visualisation of the small peritoneal implants, characteristic of advanced ovarian cancer, as shown in Figure 5. For instance, 36 h after injection, a high signal-to-noise detection (S/N > 5) of orthotopic ovarian tumours, local lymph nodes and dispersed peritoneal metastases was enabled by polymer nanoparticles, which emitted in the NIR-II range at 1060 nm. These findings support complete tumour resection in murine models [132]. Coupling with tumour-targeting peptides for fluorescence-guided surgery (FGS), showing submillimetre resolution, is facilitated by multi-functional QDs; they also offer adjustable emission and high quantum yield (38%) [133]. In relation to ovarian carcinoma, NIR-labelled molecular probes, which target G protein-coupled receptors (GnRHR) or folate receptors, have demonstrated the ability to differentiate between peritoneal lesions and neighbouring bowel or muscle. Tumour-to-intestinal ratios of more than 4 have been attainable with these probes, in addition to continued retention for periods of up to 48 h [134]. FGS are already using targeted NIR-fluorophores to help guide cytoreductive surgery of peritoneal carcinomatosis, significantly increasing lesion detection and aiding in the establishment of surgical margins; therefore, their translation into clinical practice is already ongoing [135]. Pairing NIR-fluorophore nanocarriers with therapeutic agents in nanomedicine applications enables concurrent tumour localisation and delivery, hence reinforcing theranostic function, in which visualisation and treatment of disease in the peritoneal cavity are carried out by a single nano-platform.

### 5.4. Ultrasound and Photoacoustic Imaging

Real-time and non-ionising visualisation of ovarian cancer is offered by ultrasound (US) and PA imaging, and their detection sensitivity and therapeutic effectiveness are decidedly improved by nanomedicine platforms. Typically, acoustic nanodroplets enclose a perfluorocarbon core that, under focused US, can go through acoustic droplet vaporisation, changing into echogenic microbubbles. This effect can promote local drug release while intensifying contrast. Accumulation of the nanodroplets selective to the ovarian tumour and pH-responsive conversion to microbubbles can be demonstrated by folic acid-targeted ferritin nanocages incorporating perfluoropentane (FA-FRT-PFP). Contrast-improved US imaging, together with substantial cytotoxicity, even after only low-intensity focused ultrasound activation, can be achieved using FA-FRT-PFP [136]. Profound intratumoral penetration, ROS-mediated cell killing and US/PA dual imaging that can monitor treatment response ongoingly can be exhibited by dual US-activatable nanodroplets, intended for folate receptor-positive ovarian cancer [137]. In recent studies, nanoparticles integrated with olaparib have been modified for use as ultrasound contrast agents for BRCA1 and BRCA2 (BRCA) proficient ovarian cancer, enabling integrated diagnosis and therapy by pairing PARP inhibitor delivery with contrast-enhanced ultrasound signals [138]. A complementary, completely organic PA platform, defined by strong NIR absorption, great photostability and readily achievable surface functionalisation for precise molecular targeting, can be provided by semiconducting polymer nanoparticles (SPNs) [139]. Strong contrast PA imaging and photoimmunotherapy in orthotopic and metastatic ovarian cancer models has recently been allowed by quinoidal SPNs with NIR-II absorption. This has led to the yielding of strong tumour suppression and inhibition of metastasis, while preserving a clear PA signal generated from deep pelvic lesions [140]. In tandem, dependable separation of exogenous signal from endogenous chromophores, which improved detectability of lesions and helped to guide following therapy, was enabled by a specific spectral signature PA nano-agent, engineered for ovarian cancer [141]. On the whole, activatable US/PA theranostic systems that can map, treat and vigorously monitor ovarian cancer can be established by acoustic nanodroplets and semiconducting polymer nanoparticles.

### 5.5. Multimodal Imaging Nanoplatforms

Nanosystems designed to carry out two (dual-mode) or three (tri-mode) imaging capabilities within a single construct are called multimodal imaging nanoplatforms. They were developed with the intention of overcoming limitations of traditional imaging of small peritoneal lesions, such as poor sensitivity. Additionally, they were also engineered to incorporate high-resolution structural imaging outputs, like ultrasound (US), CT and MRI, with molecular or functional information that can be provided by imaging modalities such as PA, PET and fluorescence. In a recent review about ovarian cancer nanoparticle imaging agents, the tailoring of platforms for integration into standard imaging modalities, such as fluorescence, US, PA, PET/CT and MRI, is highlighted [14]. Dual-mode anatomical-molecular imaging can be well exemplified by folate-targeted oxygen/indocyanine green lipid nanoparticles (FA-OINPs). By their implementation, both US and PA signals are enhanced in SKOV3 ovarian cancer xenografts, while under NIR laser and US irradiation, photo-sonodynamic and photothermal therapy are provided [142]. PET/CT for quantitative biodistribution and MR temperature imaging is enabled by copper sulphide nanoparticles tagged with ^64^Cu. This facilitates real-time monitoring of photothermal therapy in HeyA8 and SKOV3-ip1 ovarian cancer models, combining PET-based molecular guidance with MR-based thermometry [143]. T1-weighted MRI, fluorescence (FL), and infrared thermal (IRT) imaging in SKOV3 models are provided by biodegradable hollow mesoporous organosilica nanoplatforms (HMON@CuS/Gd_2_O_3_). This enables FL/MRI/IRT-guided mild photothermal therapy that, without systemic toxicity, can destabilise lysosomes and suppress tumour growth [144]. Phase transitional, folate-targeted perfluoropentane nanodroplets, payloaded with Fe_3_O_4_ and 10-hydroxycamptothecin (FA-HCPT-Fe_3_O_4_-PFP NDs), achieved MRI and PA imaging. After low-intensity focused ultrasound stimulated liquid-to-gas transition, a strong ultrasound contrast was generated while concurrently releasing chemotherapeutic agents in mice bearing SKOV3 xenografts. This demonstrated true MRI/PA/US tri-mode theranostics [145]. Lastly, MicroSPECT/CT-based multimodal imaging of SKOV3 xenografts is demonstrated by telodendrimer paclitaxel nanomicelles labelled with ^125^I; this guides drug delivery by quantifying tumour-selective accumulation and pharmacokinetics [146]. Collectively, these platforms show how integrated anatomical-molecular imaging in ovarian cancer nanomedicine encourages precise lesion detection, image-guided intervention, and real-time monitoring of nanotherapy effectiveness.

### 5.6. Quantitative Imaging Biomarkers

The unifying translational bridge across imaging modalities, enabling nanoparticle accumulation, retention, and therapeutic response to be compared independently of carrier composition, can be provided by quantitative imaging biomarkers. Quantitative imaging biomarkers can achieve this by delivering measurable parameters to track nanocarrier delivery and to foresee or monitor treatment response in ovarian cancer nanomedicine. Nanoparticle studies report increasing amounts of standardised parameters, such as K^trans^ and V_e_, %ID/g and tumour-to-muscle ratios. A notable example is the quantitative dynamic contrast enhanced (DCE)-MRI approach established by Nomani et al. In this study, a nano-sized MRI contrast agent was used in three ovarian cancer tumour xenograft models. Tumours were stratified for responsiveness to nanotherapeutics, and the enhanced permeability and retention (EPR) effect was estimated non-invasively by derived permeability and retention parameters, such as K^trans^ and V_e_ [147]. In relation to PET-based molecular biomarkers, Yang et al. manufactured a ^68^Ga-DOTA-c(NGR)_2_ probe, which targets CD13 in ovarian cancer xenografts. Tumour uptake as %ID/g and tumour-to-muscle ratios were quantified on microPET. PET-obtained uptake values could be recognised as biomarkers for targeted nanocarrier localisation and candidate therapy selection, due to these metrics correlating with CD13 expression and blocking experiments [148]. Reviews of nanoparticle imaging in ovarian cancer, with a focus on clinical applications, such as by Di Lorenzo et al., describe how radiolabelled liposomes, iron-oxide nanoparticles, and optical nanoprobes have been used in imaging ovarian cancer across various platforms, such as MRI, PET/SPECT, and FL. They emphasise that the quantification of imaging parameters, such as variations in signal intensity, improvement of contrast, and detectability of lesions, which enables nanoparticle distribution monitoring, and the assessment of treatment effects over time, is enabled by these nanoparticle-based approaches [149]. Combined tracking-response biomarkers are exemplified by theranostic radiolabelled liposomes. Serial SPECT/CT-derived pharmacokinetics have been connected to changes in tumour biology, involving drug resistance reversal and stemness markers, in ovarian cancer models and initial clinical work with ^188^Re-liposome. This provided imaging-based metrics of nanotherapy effectiveness [150]. Lastly, quantitative DCE-MRI and diffusion-weighted imaging (DWI) parameters, which have already been authenticated for ovarian tumour characterisation, such as K^trans^ and V_e_, are seen as easily translatable response biomarkers for future nanoparticle-based therapies in ovarian cancer [151]. Notwithstanding notable advances, the clinical translation of theranostic nanomedicine in ovarian cancer continues to be restricted by several limitations. Effective intracellular drug delivery, release of the active therapeutic component, or therapeutic response does not always correlate with imaging-detected nanoparticle accumulation; this creates a central disconnect between signal detection and efficiency. Dose mismatches between tracer-level imaging agents and clinically meaningful drug exposure deepen this challenge, in addition to spatial and temporal discordance, in which imaging reflects early vascular deposition rather than sustained intratumoral retention. Further, the reliability of quantitative imaging biomarkers as predictors of therapeutic response is reduced by pronounced inter-lesional and inter-patient heterogeneity in nanoparticle delivery, retention and release. Lastly, manufacturing and regulatory challenges that restrict batch-to-batch reproducibility and standardisation of imaging signal are introduced by the structural complexity of multimodal theranostic platforms, limiting validation of quantitative biomarkers and clinical translation. Table 4 describes multimodal imaging nanoprobes used in ovarian cancer.

Aside from lesion detection, by enabling in vivo assessment of delivery, patient stratification, and treatment adaptation, quantitative and multimodal imaging serves as a translational bridge between nanocarrier design and clinical application.

## 6. Therapeutic Nanomedicine Applications

### 6.1. Nucleic Acid Nanomedicine

The viability of siRNA nanotherapy in ovarian cancer was recognised by neutral nanoliposomes. Vigorous gene silencing decreased tumour growth, and increased paclitaxel efficiency in orthotopic models was produced by EphA2-targeting siRNA formulated in DOPC liposomes. This illustrated RNAi-mediated chemosensitisation [152]. miRNA nanomedicines build on this by aiming to reinstate tumour-suppressive networks.

In ovarian cancer models, the restored expression of miR-200c reduced the extent of metastasis and heightened sensitivity to paclitaxel. From this we can conclude miR-200c is a reasonable payload for nanocarriers that aim to reverse epithelial-mesenchymal transition (EMT) and drug resistance [153]. Additionally, PARP modulation has become nano-enabled. A polymeric formulation of olaparib, called NanoOlaparib, was able to show effective intraperitoneal exposure and tumour control in disseminated peritoneal models, whilst sustaining activity similar to a free drug. This suggested a strategy to treat late-stage disease beyond BRCA-mutant subsets [154]. Platinum resistance can be overcome by complementary multilayered liposomal nanoparticles co-loading cisplatin and a PARP inhibitor with an external hyaluronan coating for CD44; survival in HGSOC models was also improved [155]. A tumour-targeted liposomal system, delivering clustered regularly interspaced short palindromic repeats (CRISPR)/Cas9 plasmids known as CRISPR-enabled nanomedicine, is upcoming. Efficient gene editing and inhibition of growth in ovarian xenografts have been achieved by this system. This provides preclinical demonstration that nanoparticles that are non-viral can support in vivo genome editing for treatment of ovarian cancer [156].

### 6.2. Targeted Enzyme-Responsive Nanotherapies

Protease overexpression, redox imbalance, and hypoxia in ovarian tumours are made use of in enzyme- and microenvironment-responsive nanotherapies to attain spatially controlled drug activation. Selective enzymatic activation in cathepsin B-overexpressing SKOV3 and HeyA8 cells was shown by cathepsin B-specific doxorubicin prodrug nanoparticles, which were built from FRRG-DOX conjugates. Moreover, significantly improved intraperitoneal inhibition of tumour progression with decreased systemic toxicity, when compared to free doxorubicin in peritoneal carcinomatosis models, was also observed [157]. A complementary protease-activated platform is provided by matrix metalloproteinase-3 (MMP-3)-sensitive near-infrared fluorescent probes. MMP-3 stimulated signal intensification and allowed sensitive stromal imaging in an epithelial ovarian cancer xenograft. This demonstrates that MMP-responsive constructs can be adapted for image-guided nanotherapies [158]. Elevated tumour GSH is exploited by redox-responsive systems. GSH-triggered platinum release, US-mediated enhancement of tumour uptake, and strong antitumour efficiency with weakened nephrotoxicity in SKOV3 models can be achieved by GSH-sensitive Pt (IV) prodrug-loaded phase-transitional nanoparticles with a hybrid lipid-polymer shell [159]. Oxygen-poor peritoneal lesions are targeted by hypoxia-activated systems. A sphingomyelin cholesterol liposome, which encapsulated the vinblastine-N-oxide prodrug CPD100, called CPD100Li, showed more than a 9-fold increase in cytotoxicity in hypoxic conditions in ES2 ovarian cancer cells; hypoxia-activated liposomal chemotherapy for advanced ovarian cancer was further supported by satisfactory pharmacokinetics and tolerability in vivo [160].

### 6.3. Photothermal and Photodynamic Therapy

Light-activated agents are used to convert optical energy into cytotoxic heat or ROS within ovarian tumours in photothermal and photodynamic nanotherapies. Laser irradiation parameters that have a substantial influence on the therapeutic outcome of photothermal and photodynamic nanotherapies include continuous wave versus pulsed operation, duration of pulse, irradiance, and total fluence. For sustained photothermal heating, continuous-wave NIR lasers are typically used, whereas higher peak power for rapid localised heating or enhanced photodynamic activation while reducing bulk tissue damage can be enabled by pulsed irradiation. To balance effective tumour ablation or ROS generation with minimised off-target thermal injury, careful control of irradiance and fluence is necessary. IR780 is an NIR dye that can act as both a fluorophore and photothermal transducer in folate-targeted liposomal formulations. Intraoperative delineation of tumour margins is enabled by FA-IR780 nanoparticles accumulated selectively in ovarian xenograft models, and following irradiation with an 808 nm laser, the surrounding tissue was spared whilst most lesions were eradicated [161]. Local damage is further intensified by dual photothermal-photodynamic platforms; receptor-mediated uptake, strong singlet-oxygen generation and tumour elimination in ovarian cancer xenograft models, under irradiation of a single NIR laser, were demonstrated by folate-targeted Pluronic chitosan nanocapsules co-loaded with IR780 [162]. Nanocarrier co-loading with clinically used photosensitisers can also advance photodynamic efficiency. Nanostructured lipid carriers with encapsulated verteporfin can enable prolonged circulation, efficient tumour accumulation and significant inhibition of tumour growth, compared to free verteporfin, while evading the severe phototoxic side effects that can be seen with the free drug [163]. Further, PDT in an ovarian cancer ascites model was enhanced by hypocrellin-B-loaded poly(butyl cyanoacrylate) nanoparticles, which prolonged survival time compared to unconjugated drug [164]. Despite this progress, limited penetration of NIR-I light through large tumour masses restrains efficiency, driving development of NIR-II phototheranostic polymers that can more deeply penetrate tissues and could lead to better signal-to-background ratios [165].

### 6.4. Immuno-Nanomedicine

**Figure 6:**
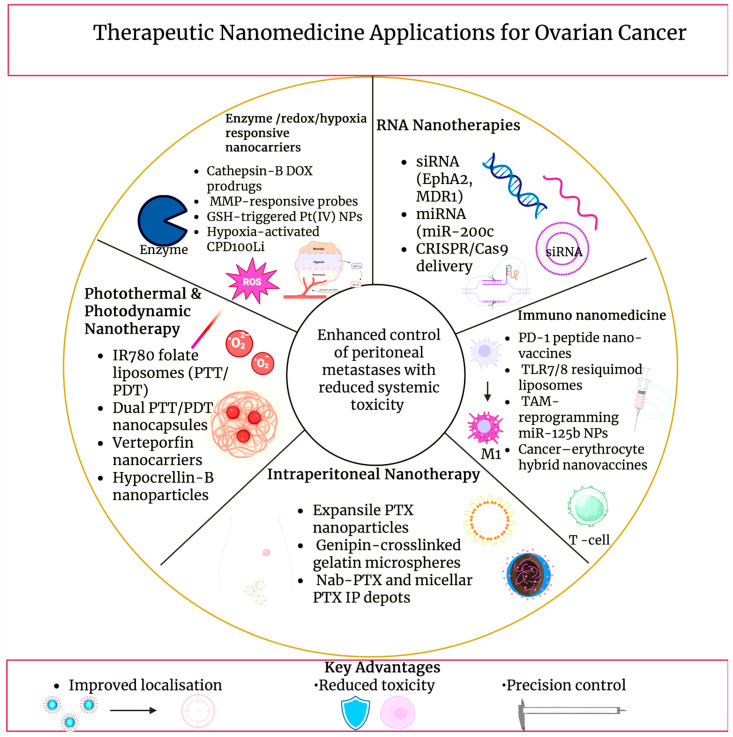
Therapeutic nanomedicine applications for ovarian cancer, illustrating representative nanocarrier-enabled treatment modalities, including photothermal and photodynamic nanotherapy, intraperitoneal drug depots, RNA-based nanotherapies, enzyme- and redox-responsive systems, and immuno-nanomedicine approaches. The schematic highlights disease-relevant therapeutic strategies aimed at improving localisation, controlling peritoneal metastases, and reducing systemic toxicity. Created in BioRender. Wilk, I. (2025) https://BioRender.com/6pds3ku.

The goal of immuno-nanomedicine is to transform the immunologically “cold” ovarian TME, which is abundant in M2-polarised TAMs but lacks effector T cells, towards a state that promotes antitumour immunity [166,167]. Only limited responses are shown by clinical checkpoint inhibitors in epithelial ovarian cancer, catalysing the need for nano-enabled strategies that enhance intraperitoneal drug delivery and immune system activation [168]. Gautam et al. designed cowpea mosaic virus nanoparticles showing an anti-programmed cell death protein-1 (PD-1) peptide for immune checkpoint inhibition. Superior tumour control and survival were demonstrated, when compared to free peptide or unconjugated virus, in an ID8 intraperitoneal model. This exemplified that multivalent nanoparticle display can strengthen local PD-1/programmed death-ligand 1 (PD-L1) inhibition, whilst simultaneously limiting systemic exposure [169]. Large anionic liposomes that migrate to peritoneal TAMs and deliver the toll-like receptor (TLR) 7/8 agonist resiquimod were engineered by Kang et al. These liposomes were successful in reprogramming TAMs towards an M1 phenotype, increasing T cell infiltration, as seen in Figure 6, and markedly enhanced the therapeutic effect of anti-PD-1 treatment in syngeneic ovarian tumours [168]. Checkpoint responses can be further amplified by antigen-directed nanovaccines. Cancer-erythrocyte hybrid membrane-camouflaged Fe_3_O_4_-ICG@IRM nanoparticles that accumulate in ID8 tumours were reported by Xiong et al. Immunogenic cell death, tumour antigen release and activation of CD8^+^ T-cell mediated immunity were triggered by NIR photothermal irradiation, demonstrating robust primary tumour inhibition and resistance to tumour rechallenge [170]. The ovarian TME can be directly remodelled by macrophage repolarising nanotherapies. Peritoneal TAMs in an ID8-VEGF model were selectively targeted by hyaluronic acid/polyethylenimine (PEI) nanoparticles encapsulating miR-125b. The TAMs were shifted to an immune-activating phenotype, and when combined with intraperitoneal paclitaxel, a reduction in ascites and VEGF levels was seen, with minimal systemic toxicity [171]. On the whole, these checkpoint-regulating, antigen-delivering and TAM-reprogramming platforms show how immuno-nanomedicine can increase immunotherapy efficiency in ovarian cancer.

### 6.5. Intraperitoneal Nanotherapy

Direct exposure of disseminated ovarian implants to nanoparticle depots is used in IP therapy to progress peritoneal disease control while minimising systemic exposure. In comparison to traditional IP solutions, nanoparticle systems are designed to remain in the peritoneal cavity, adhere to serosal surfaces and release the drug slowly, maintaining cytotoxic levels along peritoneal tumour implants dispersed across tumour-bearing peritoneal surfaces [172]. Following cytoreductive surgery, intraoperatively administered paclitaxel-loaded expansile nanoparticles notably decreased locoregional recurrence, and survival versus free paclitaxel in ovarian carcinoma models was improved. This demonstrated that intraperitoneally retained nanocarriers strengthen locoregional control of microscopic residual tumours [173]. Radiolabelled expansile nanoparticles emphasise residence time advantages; after a single IP dose, they show tumour-selective accumulation within the peritoneal cavity lasting for up to 1–2 weeks, with negligible signal in distant organs [128]. Likewise, a long-acting intraperitoneal depot was created by paclitaxel-loaded genipin-crosslinked gelatin microspheres. By this formulation, survival was prolonged, peritoneal carcinomatosis scores were lowered, and reduced ascites were seen when compared to short-acting IP controls [174]. High peritoneal-to-plasma gradients are the root of toxicity reduction. Strong inhibition of peritoneal tumour growth, with lymphatic targeting and satisfactory systemic safety, was achieved by early paclitaxel nanoparticles in ovarian models [175]. In recent times, marked antitumour efficiency and survival benefit were produced by IP administration of nanoparticle albumin-bound and micellar paclitaxel in murine ovarian xenografts. Additionally, low systemic exposure was maintained, supporting a better therapeutic index for intraperitoneal nanotherapy in ovarian cancer [176]. Table 5 summarises therapeutic nanomedicine applications in ovarian cancer.

These therapeutic outcomes emphasise how nanocarrier design can enhance efficiency while supporting clinical translation when integrated with imaging-informed patient selection and route optimisation.

## 7. Pharmacokinetics, Biodistribution and Safety Considerations

### 7.1. Physicochemical Determinants of Nanoparticle Biodistribution and Clearance in Ovarian Cancer

**Figure 7:**
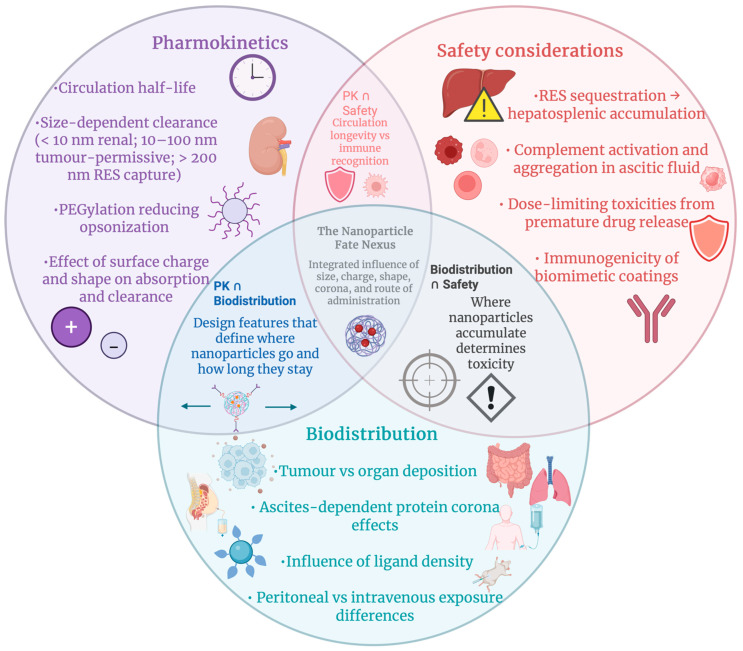
Interconnected factors controlling nanoparticle fate in ovarian cancer. Pharmacokinetics, biodistribution, and safety constraints intersect to determine circulation, tumour targeting, off-target deposition, and toxicity, forming a unified framework for designing clinically viable nanomedicines. Created in BioRender. Wilk, I. (2025) https://BioRender.com/2eoca0d.

Biodistribution and clearance in ovarian cancer nanomedicine are dictated by nanoparticle physicochemical properties. In pharmacokinetic studies, extremely small particles, with a diameter of less than 10 nm, are shown to be rapidly cleared renally, while filtration is avoided by systems with particles of a diameter of 10–100 nm, although they are still small enough to cross the vascular border and accumulate in tumours. Nanoparticles with a diameter larger than 200 nm are mostly captured by Kupffer cells in the liver and spleen [177,178]. He et al. showed that size and surface charge act together when he found that tumour uptake is maximised by nanoparticles of 150 nm diameter with a mildly negative zeta potential, while improved opsonisation and reticuloendothelial clearance can be achieved by strongly cationic or highly anionic formulations [179]. Migration, tumour penetration, and cellular internalisation are further moderated by shape, with extended circulation and altered organ tropism, compared with spheres, being exhibited with rod-like or discoidal geometries [180]. An additional bio-interface is introduced by malignant ascites in the peritoneal cavity. It is shown that ovarian cancer ascites is abundant in albumin, complement, coagulation factors and tumour-associated proteins like CA125/MUC16, by proteomic profiling, forming a complex biochemical environment for nanoparticle absorption [181,182]. Nanoparticles quickly obtain an ascites-specific protein corona, as seen in Figure 7, upon IP administration. It was established by Dakwar et al. that widespread aggregation in peritoneal and ascitic fluid can be seen by non-PEGylated cationic and anionic particles, while colloidal stability can be enhanced by PEGylation, although it can also lead to premature release of siRNA, which can reshape local pharmacokinetics [183]. Protein corona composition, as a result, controls recognition by macrophages, complement and NK cells, modulating immune clearance and tumour versus off-target uptake [184]. Therefore, ovarian cancer nanomedicines that prospered in vivo combine diameter (50–150 nm), an almost neutral or slightly negative charge, and stealth coatings with tumour-specific ligands to navigate ascites, make use of helpful coronas, and successfully achieve selective imaging and therapy [14,185].

### 7.2. Toxicity, Immunogenicity, and Reporting Standards in Ovarian Cancer Nanomedicine

A key part of translating ovarian cancer nanomedicine into the clinic is analysis of acute and chronic safety profiles. Nanoformulation can shift toxicity from acute cardiomyopathy to infusion reactions, palmar-plantar erythrodysesthesia and mucositis, whilst significantly decreasing early-onset heart failure when compared to traditional doxorubicin, as shown in clinical experience with pegylated liposomal doxorubicin (PLD) [186]. However, safety considerations that become more significant with repeated dosing and long-term exposure are introduced by the altered biodistribution and prolonged circulation that underlie these clinical benefits. For instance, it is indicated by long-term follow-ups that at very high lifetime doses, cumulative cardiotoxicity remains a concern, although if cardiac risk factors and function are monitored carefully, PLD can frequently be given without a strict upper limit [187]. Past organ toxicity, immunogenicity can be triggered by nanomedicines. Nano-specific adverse events like complement activation-related pseudoallergy (CARPA) and other infusion reactions that are more frequent with certain liposomal and micellar products than with their standard formulations can be revealed by pharmacovigilance data. For nanomedicine-based products that are intended for use in oncology, including ovarian cancer regimens, structured in vitro and in vivo assessments of cytokine release, complement activation, hypersensitivity and unintended immune suppression or stimulation are advised by immunotoxicology guidelines [188]. Highly important for younger patients is reproductive toxicity, though it is poorly characterised. It is shown by experimental and epidemiological analyses that various nanoparticles can cross placental and blood-testis barriers, disturb spermatogenesis by accumulating in gonads, and disrupt folliculogenesis and embryo development through oxidative stress and DNA damage, therefore supporting rigorous pregnancy exclusion, contraception counselling and prudent dose translation from preclinical models to ovarian cancer trials [189]. Though, commonly used preclinical models often fail to capture the fibrotic, heterogenous, and ascites-rich peritoneal environment that has a critical influence on nanoparticle biodistribution, toxicity, and therapeutic response in patients, limiting their predictive value. Vital for interpreting these risks and their translational relevance is high-quality reporting. The MIRIBEL checklist outlines minimum information for bio-nanomaterial experiments so that toxicity and efficiency in ovarian cancer models can be reproduced and compared. It requires detailed nanoparticle physicochemical characterisation, describing size, charge, corona and stability, dosimetry, biological models and statistics [190]. The ARRIVE 2.0 guidelines order transparent reporting of animal strain, randomisation, blinding, sample size reasoning and humane endpoints to reduce bias and advance translatability for in vivo ovarian cancer nanomedicine studies [191]. These frameworks together reinforce best practice in nano-bio characterisation and safety assessment for imaging and therapeutic applications in ovarian cancer. Table 6 describes pharmacokinetic, biodistribution and safety signatures of representative nanomedicines in ovarian cancer.

## 8. Clinical Translation and Regulatory Perspective

### 8.1. Clinical Progress, Therapeutic Outcomes, and Stratification Challenges in Ovarian Cancer Nanomedicine

**Figure 8:**
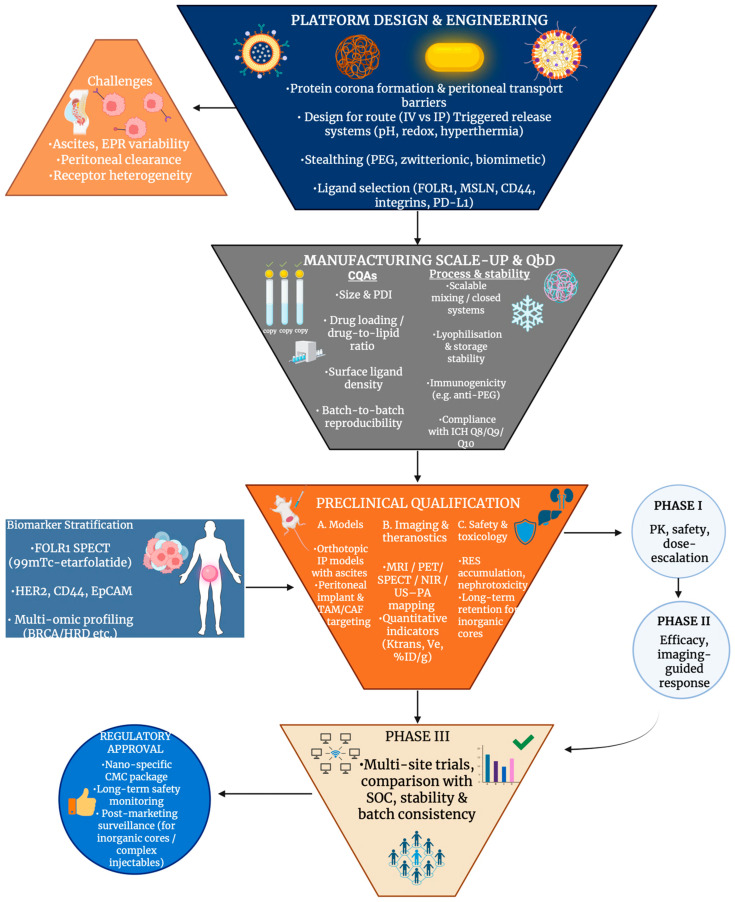
Integrated clinical translation funnel for ovarian cancer nanomedicine, illustrating the sequential progression from platform engineering and manufacturing quality control through preclinical qualification, biomarker-guided patient stratification, and early-to-late clinical trials, culminating in regulatory approval. Created in BioRender. Wilk, I. (2025) https://BioRender.com/bdd729t.

PLD and nanoparticle taxanes lead clinical nanomedicine trials in ovarian cancer, while RNA-loaded lipid systems remain mostly preclinical. In MITO-2 first-line carboplatin-PLD was at least as effective as carboplatin-paclitaxel, with both showing similar survival, although carboplatin-PLD demonstrated lower neurotoxicity and alopecia, accompanied by increased risk of haematologic adverse effects [192]. In relapse due to platinum resistance, PLD used alone, again with a distinct toxicity profile, was established to be no less effective than gemcitabine or topotecan in phase III trials [193]. PLD has been fixed in combinations with trabectedin and/or bevacizumab. When carboplatin-gemcitabine-bevacizumab was compared against carboplatin-PLD-bevacizumab, better outcomes were seen with the latter in platinum-eligible recurrence with acceptable safety [194], and trabectedin-PLD is supported as a substitute option by phase III data [195]. In refractory disease, nanoparticle albumin-bound paclitaxel (nab-PTX) has shown notable standalone activity [196], and due to reports from phase I studies showing advantageous pharmacokinetics and controllable dose-limiting toxicities, as seen in Figure 8, it is being repositioned for intraperitoneal or aerosolised delivery (PIPAC) in peritoneal metastases [197,198]. Clinical translation of siRNA nanomedicine is at earlier stages of advancement. Though ongoing preclinical evaluation is supported by DOPC-based EphA2-siRNA liposomes (EPHARNA) completing good laboratory practice (GLP) safety studies in mice and other non-human primates [199]. Vigorous prolongation of survival in orthotopic models, but no late-phase human data yet, is shown by multiple lipid and hybrid LNP platforms, which target PLK1/elF3c, Parp1 or CD44 positive cells [200,201]. Instead of radically transforming outcomes, generally, nanomedicines modestly prolong progression-free survival and improve tolerability. A lack of prospective stratification by target expression, such as FOLR1, EphA2 and CD44, along with integration of companion imaging or biopsies being limited, leads to ‘nanotargets’ being rarely used to select patients for specific nanomedicine treatments; this signifies a central limitation and represents an area of improvement in upcoming ovarian cancer nanomedicine trials.

### 8.2. Manufacturing, Stability and Regulatory Requirements for Ovarian Cancer Nanomedicines

Strict chemistry, manufacturing, and control (CMC) information is required for manufacturing nanomedicines for ovarian cancer. Tight control of significant quality attributes, such as size, polydispersity and surface charge, is required for complex structures such as liposomes, polymeric nanoparticles and micelles, to guarantee reproducibility between batches and foreseeable pharmacokinetics, as stated in reviews of translational applications of nanomedicine [202]. Small differences in mixing, exchange of solvents or lyophilisation can change ovarian cancer formulations such as pegylated liposomal doxorubicin [203] or nanocomplexes loaded with cisplatin [204]. Parenteral nanomedicines have to be sterile; however, destabilisation of nanocarriers may occur through thermal or radiation sterilisation. Consequently, cost is often increased by aseptic processing and sterile filtration being required, which demands robust stability profiles over storage and after reconstitution [205]. Aggregation, drug leakage and matrix oxidation are features of long-term physiochemical stability that hinder ovarian cancer nanoformulations from entering clinical trials [206]. Together with complex analytics, these CMC and stability requirements raise manufacturing costs, which limit access regardless of clinical benefit in recurrent disease [207]. Furthermore, the clinical progression of complex, multi-element nanomedicines is significantly limited by these constraints, as reliable, reproducible nano-formulations with well-defined critical quality attributes are favoured for large-scale production and regulatory approval. Ovarian nanomedicine regulatory frameworks build on general drug regulations but also include specific FDA and EMA expectations for nanomedicines. The need for thorough characterisation, risk-based EMC strategies and post-marketing surveillance is emphasised by FDA- and EMA-focused reviews, which reference nanotechnology guidance and EMA position papers on liposomes and other nanoproducts [208]. Regulators are more frequently expecting co-validation of imaging or soluble biomarkers, like CA-125 and emerging multiplex panels, for theranostic ovarian platforms [209], evaluated in conjunction with the nanomedicine consistent with the broader shift toward imaging-based companion diagnostics and biomarker-guided theranostics in ovarian cancer [210]. Table 7 summarises the clinical translation of ovarian cancer nanomedicines.

## 9. Future Perspectives and Challenges

**Figure 9:**
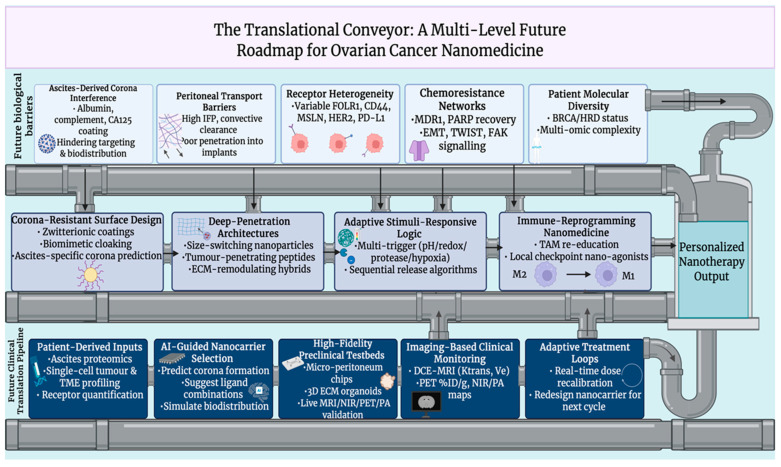
A multi-level “translational conveyor” illustrating how biological barriers in ovarian cancer guide the development of next-generation nanomedicine engineering strategies and feed into an iterative clinical translation pipeline, ultimately enabling personalised nanotherapeutic design. Created in BioRender. Wilk, I. (2025) https://BioRender.com/7e0wznz.

The integration of multi-omics profiling, such as genomics, transcriptomics and proteomics, with AI-based modelling is being increasingly relied on in ovarian cancer precision nanomedicine to stratify patients, predict drug response and direct nanocarrier design. Signatures associated with early detection and prognosis of ovarian cancer can be identified through AI frameworks trained on molecular and imaging biomarkers, although their clinical use is hindered by small, heterogenous datasets and a lack of standardised image and omics workflows [211]. In theory, these models could be coupled to nanoparticle libraries, and based on individual tumour profiles and longitudinal radiomics data, tailored ligand combinations, payloads and dosing schedules could be selected for each patient. At the centre of “imaging-targeting-therapy” concepts in ovarian cancer are multifunctional theranostic platforms; for example, a comprehensive review about imaging and therapy of ovarian cancer: Clinical applications of nanoparticles describe that nanotheranostic nanoparticles, which integrate imaging and drug delivery, function in a single formulation and successfully monitor drug distribution profiles and pathological processes longitudinally in ovarian cancer patients, thus offering a promising approach for diagnostics and therapy [149]. For instance, CA125-targeted radiolabelled antibodies, folate-decorated liposomes and photodynamic nanoparticles for ovarian cancer. Plasmodic and exosome-based sensors are examples of emerging nanotechnologies for early detection that are being adapted to recognise proteins specific to ovarian cancer and nucleic acids in blood, urine or ascites [212]. Due to HGSOC being “immune cold”, TME and immune modulation have developed into essential design criteria for nanomedicine. Kaur et al. describe nanoparticles that reprogram TAMs, deliver TLR agonists like resiquimod to peritoneal TAMs, boost infiltration of CD8^+^ T-cells and synergise with PD-1/PD-L1 blockade [213]. Comparable future strategies also apply to cytokine-loaded platforms, such as IL-12 nanoparticles, and redox- or pH-sensitive micelles that exploit hypoxia, acidosis or high GSH levels in the peritoneal cavity to stimulate localised drug release and turn “cold” lesions into “hot” ones, as seen in Figure 9. Balanced adjustment of size, charge and stiffness to favour intraperitoneal retention and stromal penetration is highlighted in reviews of TME-responsive nanomedicines [214,215].

However, clinical translation is slowed down by inconsistent regulatory frameworks and non-standardised datasets. Overall, in nanomedicine, the lack of reliable definitions, incomplete physiochemical characterisation and unpredictable safety endpoints across authorities are highlighted by regulators [216]. Coordinated international standards bodies and the European Medicines Agency (ISO/CEN-EMA) efforts, common test panels for nanoparticle identity, stability and interoperable data formats which link preclinical, imaging and clinical outcome data, are called for in a proposed standardisation roadmap [217]. Simultaneously, before AI-based decision tools for ovarian cancer can guide regulatory-grade precision nanotherapy, they must meet developing requirements for transparency, bias control and external validation [211].

Ovarian cancer nanomedicine could be redesigned by several emerging technologies. Scalable, exosome-like carriers with inherent tumour tropism can be offered by immune cell-derived exosome mimetics (IDEM), which can deliver small RNAs or drugs to resistant cells in ovarian models and could overcome some manufacturing obstacles of natural exosomes [218]. Enzyme- and pH-responsive platforms for drug and immunomodulator delivery can be demonstrated by self-assembling peptide nanostructures that can also be used as 3D matrices that replicate the multicellular ovarian TME for screening nanotherapeutics [219]. Lastly, strong imaging contrast or photothermal properties with tuneable clearance are combined by biodegradable metallic nanocarriers, such as gold or zinc oxide systems, which are engineered for controlled dissolution. This signifies a promising direction for safer theranostic platforms in ovarian cancer [220].

## 10. Conclusions

The diagnostic and therapeutic landscape of ovarian cancer is being rapidly reshaped by nanomedicine, presenting practical solutions to persistent challenges such as late detection, drug resistance, peritoneal dissemination, and dose-limiting toxicity. Nanoparticles reliably demonstrate the ability to improve drug pharmacokinetics, advance tumour-selective delivery, and facilitate high-resolution multimodal imaging across lipid-based, polymeric, inorganic, hybrid, and biomimetic platforms, which together form the basis of highly precise and adaptive ovarian cancer care. Notably, nanocarriers are progressively designed to navigate the ovarian TME and its biological complexities, such as hypoxia, dense ECM, ascites and expression of heterogenous receptors, by which the effectiveness of systemic chemotherapy was historically hindered. Innovative nanomedicines achieve deeper tumour penetration, maintain stable intraperitoneal residence, and reduce systemic toxicity by integrating targeting ligands, stimuli-sensitive release designs and stealth strategies, thus widening the therapeutic window against advanced ovarian cancer.

A particularly transformative development is represented by theranostic platforms. Clinicians can visualise tumour load, guide surgical resection, and track treatment response in real time by uniting imaging and therapy into a single platform, such as MRI-trackable liposomes, radiolabelled polymeric particles and photoacoustic-sensitive nanodroplets. As peritoneal implants are often microscopic, widely disseminated and hard to detect with standard imaging, these capabilities address a critical need in ovarian cancer that is addressed by theranostic platforms.

Although clear progress has been made, clinical translation is still limited. Based on the evidence synthesised in this review, in order to accelerate clinical translation, several priorities must be addressed. A primary priority is the identification of patients who are most likely to benefit from targeted or theranostic nanomedicines using molecular imaging and companion diagnostics by emphasising biomarker-guided patient stratification in future research. A further priority is to allow personalised dosing, early response assessment, and adaptive treatment strategies by expanding the integration of quantitative imaging biomarkers into nanomedicine development. An additional priority is to place greater focus on intraperitoneal delivery systems and ascites-resistant designs, which remain under-represented in clinical trials although they are uniquely suited to the dissemination pattern of ovarian cancer. A further priority is to advance the standardisation of nanocarrier characterisation, scalable manufacturing processes, and regulatory alignment in order to improve reproducibility and enable regulatory approval. Lastly, a promising direction to overcome chemoresistance and immune suppression in advanced disease is represented by integrating immunotherapy, gene-based approaches and stimuli-responsive platforms. Complementary advances in immunonanomedicine further support this strategy by aiding TAM reprogramming, improving T-cell infiltration and promoting synergistic checkpoint blockade in immune-cold HGSOC.

To conclude, the future impact of nanomedicine in ovarian cancer will rely not only on ongoing material and engineering innovation but also greatly on strategic integration with molecular profiling, quantitative imaging, and clinically relevant delivery routes. In order to translate nanotechnology from experimental platforms into operative, patient-centred solutions for ovarian cancer management, addressing these challenges will be essential.

## Figures and Tables

**Table 1:** Key surface biomarkers and molecular targets relevant to nanomedicine in ovarian cancer [11,29,30,31,32,33,34,35,36].

Target/Receptor	Expression Pattern in Ovarian Cancer	Biological/Pathophysiological Role	Rationale for Nanomedicine Targeting	Example Nanomedicine Approaches
Folate Receptor-α (FOLR1/FR-α)	Highly overexpressed in HGSOC, limited in normal tissues	Drives folate uptake Associated with tumour aggressiveness	- Abundant and selective expression allows high-affinity binding for drug-loaded carriers	- Folate-decorated liposomes, micelles, MRI/fluorescent nanoprobes - Patient selection via FR-specific SPECT (99mTc-etarfolatide)
Mesothelin (MSLN)	Overexpressed in serous ovarian carcinoma and peritoneal implants	Mediates peritoneal adhesion through MSLN-MUC16 binding Facilitates dissemination	- Accessible on tumour surface and peritoneal metastasis	- Anti-MSLN antibody-guided nano micelles, targeted nanoparticles enhancing tumour uptake and reducing toxicity
MUC16/CA-125	Overexpressed on tumour cells and shed into ascites	Promotes metastasis via mesothelial binding Modulates immune evasion	- Highly characteristic ovarian cancer marker	- Anti-MUC16 nanomicelles - Aptamer-drug conjugates - Fluorescence-guided constructs
EpCAM	Strong epithelial marker and linked to chemoresistance	Supports adhesion, proliferation and survival	- Present on circulating tumour cells - Accessible on residual disease	- EpCAM magnetic particles (MICAD probes) - Targeted imaging and drug delivery systems
CD44	Elevated on tumour and cancer stem-like cells	Hyaluronan receptor driving metastasis, stemness and chemoresistance	- Enables HA-based or dual ligand targeting - Ideal for reaching resistant subpopulations	- HA-PLGA nanoparticles (PTX/FAK-siRNA) - MDR1-siRNA systems - CD44-guided siRNA carriers
Integrins (αvß3, αvß5)	Overexpressed on tumour cells and angiogenic vasculature	Promote invasion, angiogenesis, and metastatic survival	- Well-validated ligands, e.g., RGD peptides, allow tumour-endothelium targeting	- RGD-labelled liposomes, siRNA carriers, photothermal systems, intraperitoneal probes
PD-L1	Variably expressed, upregulated in immune-cold ovarian tumours	Suppresses T cell activation Drives immune evasion	- Enables integration of immune-nanomedicine and theranostic immune-responsive systems	- PD-L1-targeted polymer conjugates - Checkpoint enhancing nanocarriers for TAM modulation
Folate Receptor-ß (FR-ß) on TAMs	Selectively expressed on TAMs	Sustains immunosuppressive microenvironment	- Allows TAM-specific drug or RNA delivery	- FR-β-targeted macrophage nanotherapies - TAM-reprogramming platforms
Tumour endothelial marker 1 (TEM1)	Expressed on tumour vasculature	- Supports angiogenesis and vascular remodelling	- Enables vascular targeted drug delivery	- TEM1-directed active targeting paired with stealth coatings

**Table 2:** Key nanocarrier designs for ovarian cancer: composition, release triggers and performance [79,81,82,83,84,85,86,87].

Nanocarrier Platform	Core Composition and Architecture	Primary Release Triggers	Key Advantages for Ovarian Cancer	Limitations/Engineering Challenges	Clinically Relevant and Representative Examples
Lipid-Based Systems: Liposomes, LNPs, and Thermosensitive Liposomes	Phospholipid bilayers - PEG-lipids - Ionisable lipids - Cholesterol	pH (endosomal), heat (thermosensitive), enzymatic	- Clinically validated - Long circulation (PEG) - Reduced cardiotoxicity - Ideal for siRNA, mRNA - Adaptable to folate, RGD or antibody targeting	- Accelerated blood clearance - Stability issues during sterilisation/lyophilisation - Protein corona in ascites - Ligand density balance	- PLD/Doxil - Thermosensitive liposomes - DOPC-based EphA2 siRNA carriers (EPHARNA)
Polymeric Nanoparticles: PLGA, PEG-PLA Micelles, PAMAM, and PDCs	- Biodegradable PLGA - PEG-PLA micelles - Dendrimers - Polymer–drug conjugates	pH-triggered PEG shedding - Redox (GSH) - Enzyme, e.g., cathepsins, -MMPs - ROS	- High stability - Tunable surface chemistry - Dual loading (drug + siRNA/miRNA) - Strong peritoneal retention - Proven chemoresistance reversal	- Slower degradation - Acidic degradation byproducts - Manufacturing variability - Entrapment heterogeneity	- HA-PLGA PTX+FAK siRNA - PEG-PLA micelles - Redox-reactive PAMAM - PD-L1 polymer–drug conjugates
Inorganic Systems: Gold, Iron Oxide, Silica, and Quantum Dots	- Gold nanoshells/rods - SPIONs - Mesoporous silica - QDs	Photothermal (NIR), magnetic hyperthermia, pH, enzyme	- Strong imaging contrast (MRI/PA/CT) - Plasmonic heating for ablation - Large pore volumes (MSNs) for loading - Precision tumour mapping	- Non-biodegradable cores (some types) - Long-term accumulation risk - Potential ROS-mediated toxicity - Scale-up challenges	- Gold rods/shells for PTT - 166Ho-MSNs - SPIONs for MRI tracking - MUC1-QD drug conjugates
Hybrid Nanoplatforms: Core–Shell, Magnetic–Silica, and Organic–Inorganic	- Multilayer structures combining lipids, polymers, and metals/silica	Multi-stimuli: pH, thermal, magnetic, and enzymatic	- Enables tri-modality imaging - Theranostic precision - Controlled spatiotemporal release - Improved ascites navigation via coatings	- Complex synthesis - Higher regulatory burden - Difficult stability characterisation - Batch reproducibility issues	- Magnetic MSNs - Plasmonic silica shells - CuS multimodal platforms - FA-HCPT-Fe_3_O_4_ perfluoropentane nanodroplets
Biomimetic & Stealth Systems: Cell-Membrane Coated and Zwitterionic	- RBC-, platelet-, leukocyte-, and cancer cell–derived - Membranes zwitterionic polymers	pH, redox, enzyme (protected by antifouling)	- Immune evasion - Homotypic targeting - Prolonged intraperitoneal residence - Strong anti-fouling in ascites	- Membrane purity/consistency - Complex manufacturing - Potential immunogenicity if derived from tumour cells	- CD47-bearing cloaked nanoparticles - UCST zwitterionic nanogels - Membrane-coated hybrids for dual targeting

**Table 3:** Molecular targetability matrix linking receptor-ligand systems to nanocarrier selection and clinical decision-making in ovarian cancer [55,113,114,115,116,117,118,119,120,121,122,123,124].

Biological Niche	Receptor (Target)	Ligand/Binding Moiety	Rationale & Expression Pattern	Nanocarrier Fit	Therapeutic/Imaging Advantages	Clinical Relevance
Tumour cell surface	Folate Receptor-α (FOLR1)	Folate, anti-FOLR1 antibodies	Highly overexpressed in HGSOC, restricted in normal tissue, excellent tumour: normal ratio	Liposomes, micelles, SPIONs, and NIR probes	- Enhances uptake of chemo, siRNA - Supports PET/SPECT companion imaging, e.g., 99mTc-etarfolatide	- Clinically validated for patient stratification - FR-targeted drug conjugates in trials
	HER2	Trastuzumab fragments, HER2 peptides	- Subset of ovarian tumours show HER2 and phenotype - Especially relevant in chemo-resistant disease	Iron oxide NPs and gold nanoshells	MRI contrast, photothermal ablation, and cisplatin co-delivery	- Present in molecular imaging agents - HER2-guided theranostics emerging
	EpCAM	EpCAM antibodies, aptamers	- Strongly expressed on epithelial tumour cells and CTCs - Chemoresistance marker	Magnetic NPs and polymeric carriers	- Capture of CTCs, residual disease imaging - CTC-guided nanotherapy potential	- Used in MICAD imaging probes - Strong translational potential
	CD44 (CSC marker)	Hyaluronic acid (HA)	- Marks stem-like and chemo-resistant populations - Abundant in peritoneal metastasis	HA-PLGA, micelles, and dendrimers	- Selective delivery of siRNA (MDR1, FAK), PTX, and PARP inhibitor co-loading	- Important for resistance reversal - Multiple preclinical successes
Metastasis/Peritoneal adhesion	Mesothelin (MSLN)	Anti-MSLN antibodies, peptides	Mediates mesothelial adhesion with MUC16 and is the core driver of peritoneal spread	Antibody-NP conjugates and polymeric micelles	- Enhances uptake in metastatic nodules - Reduces systemic toxicity	- Used in MSLN-targeted drug conjugates clinically
	MUC16/CA-125	Aptamers, anti-MUC16 antibodies	- Defining ovarian cancer antigen - Shed into ascites - Drives metastasis and immune evasion	Aptamer-drug conjugates and NIR probes	Enables fluorescence-guided surgery and selective delivery	- Highly characteristic biomarker - Used clinically for disease monitoring
	Integrins (αvß3/avß5)	RGD peptides	- Overexpressed on tumour cells and angiogenic endothelium-Invasive phenotype marker	Liposomes, siRNA carriers, and photothermal systems	Dual targeting, tumour and angiogenic vasculature, and intraperitoneal imaging	- Widely Validated-Strong imaging utility (PA, NIR, and MRI)
Stromal & angiogenic targets	TEM1 (Tumour endothelial marker-1)	Anti-TEM1 antibodies	- Expressed on tumour vasculature - Limited healthy expression	Stealthed liposomes and polymer NPs	Enables vascular targeting and improved penetration into dense peritoneal nodules	Emerging vascular target with expanding utility
	CD44 on endothelium	Hyaluronic acid	Supports endothelial remodelling and metastasis	HA-siRNA carriers	siRNA delivery to tumour endothelium, e.g., FAK and MDR1	Bridges tumour and vascular targeting
Immune microenvironment	PD-L1	PD-L1 antibodies, peptides	- Variably expressed - Drives immune suppression in “cold” HGSOC	Polymer–drug conjugates and immune-NPs	- Enables immune-responsive theranostics - Synergistic checkpoint therapy	- Important for TAM/T-cell reprogramming - Relevant to immune-nanomedicine
	Folate receptor-ß (TAMs)	Folate	Selectively expressed on immunosuppressive M2-TAMs	TAM-targeted nanocarriers and macrophage-reprogramming systems	TAM re-education and enhanced chemo-immune synergy	Ideal for cold-to-hot tumour conversion
	MISRII/Other GPCRs	Antibodies, peptides	Overexpressed in subsets of ovarian cancer	Radiolabelled NPs and PET tracers	Enables PET-based molecular imaging and candidate selection	In preclinical imaging and imaging-therapy pairing

**Table 4:** Multimodal imaging nanoprobes in ovarian cancer: modalities, targets and theranostic outcomes.

Imaging Modality	Nanoprobe Platform	Target/Mechanism	Model and Key Findings	Potential Clinical Utility
MRI (T1/T2-based)	Folate-decorated Fe_3_O_4_ SPIONs	FOLR1-overexpressing ovarian tumours	- Orthotopic intraperitoneal models - Strong negative T2 contrast enabling detection of peritoneal implants	Imaging and tumour mapping of micrometastases
	Cisplatin-tagged HER2-targeted iron oxide NPs	HER2-positive ovarian tumours	Enhanced MRI contrast and intratumoral NP accumulation - Validated in HER2+ xenografts	MRI-guided cisplatin delivery
	Dual-Gd liposomes with indocyanine green (ICG)	- No receptor targeting - Vascular or peritoneal uptake	- Strong T1 enhancement - Improved delineation of intraperitoneal nodules - NIR signal complementary for surgery	MRI and NIR surgical guidance
PET/SPECT	Radiolabelled expansile nanoparticles, e.g., ^99^mTc ENPs	Peritoneal tumour surface binding	- 30% injected dose in IP tumours - Clear IV vs. IP biodistribution mapping in advanced models	PET/SPECT-guided route optimisation for therapy
	^89^Zr-labelled anti-MISRII antibodies	MISRII	- Strong tumour-to-background ratios - Non-invasive PET lesion mapping in EOC xenografts	Companion diagnostic for radionuclide therapy
	^64^Cu- or ^99^mTc-labelled polymer/liposome carriers	Passive and ligand-guided targeting	- Defined PK and tumour accumulation - Supports individualised nanomedicine dosing	Longitudinal whole-body NP tracking
Optical/NIR-I/NIR-II	NIR-II polymer nanoparticles (1060 nm emission)	Passive tumour uptake and enhanced retention	- High S/N > 5 - Visualised lymph nodes and dispersed peritoneal metastases 36 h post-injection	- Sub-mm lesion detection - Improved surgical resection
	Multi-functional QDs, e.g., MUC1 aptamer–QD	MUC1-positive tumour cells	- High quantum yield for FGS - Accurate tumour boundary delineation	Imaging and targeted DOX delivery
	NIR-labelled GPCR-targeted probes (GnRHR, FR)	GnRHR and FOLR1	- Tumour-to-intestine ratios > 4, stable ≥ 48 h - Accurate peritoneal lesion differentiation	Image-guided cytoreductive surgery
Ultrasound and acoustic imaging	Folate–ferritin nanocages with perfluoropentane	- FOLR1 - pH-activated droplet-to-bubble transition	- Strong US contrast after activation - Increased cytotoxicity with low-intensity focused ultrasound	US-triggered drug release and tumour imaging
	Dual US-activatable nanodroplets	FOLR1 or integrin-marked tumours	- Deep penetration and ROS-mediated killing - PA/US dual tracking of treatment response	Photoacoustic, US imaging and therapy
Photoacoustic (PA)	Semiconducting polymer nanoparticles (SPNs)	- NIR-II absorbing - Non-targeted or functionalised	- Strong PA contrast for deep pelvic lesions - Robust tumour suppression and metastasis inhibition with photoimmunotherapy	PA imaging and photothermal/immune synergy
	Spectrally encoded PA nano-agents	Ovarian tumour chromophores	- Enables separation of tumour signals from endogenous background - Enhances detectability of lesions	PA imaging and guided therapy
Tri-modal platforms	FA-HCPT-Fe_3_O_4_-PFP nanodroplets	FOLR1	- MRI/PA/US imaging - LIFU-triggered gas transition - HCPT release - Strong tumour suppression in SKOV3 xenografts	Full tri-mode theranostics: diagnosis, activation and therapy
	HMON@CuS/Gd_2_O_3_ hybrid	Passive accumulation and Gd enhancement	- FL/MRI/IRT guidance - Lysosomal disruption and mild photothermal therapy without systemic toxicity	Triple imaging and synergistic photothermal therapy
	^64^Cu–CuS nanoagents	PET, PA and PTT	- Enables PET biodistribution mapping and MR-temperature imaging for real-time PTT feedback	PET, MR, and PA guided photothermal ablation

**Table 5:** Therapeutic nanomedicine applications in ovarian cancer: summary of cargo, nanoplatforms, and preclinical efficiency outcomes.

Therapeutic Category	Drug/Cargo	Nanoplatform	Mechanism/Target	Preclinical Efficiency and Translational Considerations
RNA nanotherapeutics (siRNA, miRNA, and CRISPR)	EphA2 siRNA	DOPC-neutral liposomes (EPHARNA)	Gene silencing of EphA2	- Decreased tumour growth - Increased PTX sensitivity - Strong orthotopic inhibition
	MDR1 siRNA	HA-decorated carriers	CD44 targeting of chemoresistant cells	- Reversal of PTX resistance - Selective killing of CD44+ subpopulations
	miR-200c	Polymeric nanoparticles	- EMT reversal - Metastasis suppression	- Decreased metastasis - Increased PTX responsiveness - Restored tumour suppression pathways
	CRISPR or Cas9 plasmid	Tumour-targeted liposomal system	In vivo genome editing	- Tumour growth inhibition demonstrated in xenografts - First proof of concept
Chemotherapy-enhancing nanocarriers	Paclitaxel (PTX)	HA-PLGA nanoparticles	Dual cargo PTX and FAK siRNA	- Reverses chemoresistance - Enhanced peritoneal retention - Strong tumour inhibition
	Nab-paclitaxel	Albumin-bound nanoparticles	Improved PK and tumour delivery	- Marked antitumour activity - Low systemic toxicity - Effective IP use in xenografts
	Cisplatin and PARP inhibitor	Hyaluronan-liposomal hybrid	- CD44 targeting - Platinum resistance reversal	- Increased survival in HGSOC models - Strong synergy in recurrent disease
Enzyme/pH/Redox-responsive nanotherapies	DOX–FRRG prodrug	Cathepsin B–activated NPs	Protease-triggered DOX release	- Significant IP tumour suppression - Decreased systemic toxicity vs. free DOX
	Pt (IV) prodrug	GSH-responsive hybrid nanoparticles	Redox-triggered platinum release	- Tumour apoptosis - Decreased nephrotoxicity - US-enhanced uptake
	Vinblastine-N-oxide (CPD100)	Hypoxia-activated liposomes (CPD100Li)	Hypoxic peritoneal lesion targeting	- >9× cytotoxicity in hypoxia - Favourable PK - Effective in late-stage models
Photothermal/photodynamic nanotherapies	IR780 dye	Folate-targeted liposomes	NIR PTT and tumour imaging	- Complete or near-complete tumour ablation - Spared healthy tissue
	IR780 and PDT agents	Folate–Pluronic–chitosan nanocapsules	Dual PTT–PDT activation	- Strong singlet oxygen generation - Full tumour elimination in xenografts
	Verteporfin	Nanostructured lipid carriers	Prolonged circulation and deep tumour uptake	- Superior tumour inhibition vs. free verteporfin - Reduced phototoxicity risk
	Hypocrellin B	PBCA nanoparticles	Enhanced PDT	- Increased survival - Potent inhibition of ovarian ascites progression
Immuno-nanomedicine	PD-1 peptide	Cowpea mosaic virus nanoparticles	Multivalent immune checkpoint inhibition	- Strong tumour control - Increased survival vs. free peptide or virus alone
	TLR7/8 agonist Resiquimod	Large anionic liposomes	TAM reprogramming and checkpoint synergy	- TAM M2 to M1 switch - Increased T-cell infiltration - Boosted response to anti-PD-1 therapy
	miR-125b	HA-PEI nanoparticles	TAM phenotype shift	- Decreased ascites - Decreased VEGF - Effective with IP paclitaxel - Minimal systemic toxicity
Intraperitoneal nanotherapies for peritoneal metastases	Paclitaxel	Expansile nanoparticles (IP)	Slow release and tumour adherence	- Major reduction in recurrence - Significantly improved survival post-surgery
	Paclitaxel	Genipin-crosslinked gelatin microspheres	Long-acting IP depot	- Decreased carcinomatosis scores - Prolonged survival - Decreased ascites
	Paclitaxel (nanomicellar or Nab-PTX IP)	Micelles/albumin NPs	High peritoneal:plasma ratio	- Strong tumour suppression - Minimal systemic exposure led to superior therapeutic index

**Table 6:** Pharmacokinetic, biodistribution, and safety signatures informing clinical use of representative nanomedicines.

Nanomedicine Type/Example	Key Physicochemical Properties	Pharmacokinetic Profile	Biodistribution Characteristics	Safety and Immunogenicity Notes
PEGylated Liposomal Doxorubicin (PLD)	80–100 nm, PEGylated, and neutral/mildly negative charge	- Long circulation - Reduced Vd - Delayed clearance - Stable plasma exposure curve	- Tumour-selective accumulation via EPR - Limited peritoneal penetration when IV - Uptake in RES organs moderate	- Reduced cardiotoxicity vs. DOX - Infusion reactions and palmar–plantar erythrodysesthesia possible - Long-term cardiotoxicity low with monitoring
DOPC-siRNA Liposomes, e.g., EphA2 siRNA	- 70–120 nm - Neutral surface	- Sustained gene-silencing exposure - Prolonged IP retention - Minimal systemic absorption when IP	- High peritoneal tumour uptake - Low liver/spleen accumulation - Efficient penetration of small implants	- Well tolerated in mice and primates (GLP studies) - Minimal cytokine activation - Low complement activation
Expansile Nanoparticles (IP paclitaxel ENPs)	- 100–200 nm - Expandable polymer - Hydrophobic interior	- Slow intraperitoneal release led to long-acting PK - High local drug concentration for days–weeks	- Longer than 1–2 weeks tumour-associated signal - Negligible systemic distribution - Strong serosal adhesion improves depot effect	- Low systemic toxicity - Minimal off-target organ exposure - No major inflammatory response reported
Radiolabelled Liposomes/Polymeric NPs, e.g., ^99^ᵐTc or ^64^Cu-labelled	- 80–150 nm - PEGylated	- Allows full PK quantification - Circulation half-life extended - Route (IV vs. IP) dramatically alters PK curves	- PET/SPECT: High peritoneal tumour uptake with IP dosing - IV dosing led to RES uptake in liver/spleen and moderate tumour accumulation	- Useful for identifying off-target accumulation - Radiation dose manageable - Generally safe within preclinical range
Iron Oxide Nanoparticles (SPIONs)	- 10–30 nm cores - PEG or ligand-coated	- Moderate circulation - Faster clearance than liposomes - Predictable magnetic tracking	- Accumulation in tumour and strong liver/spleen uptake - Folate-targeted versions enhance tumour: organ ratio	- Potential ROS-mediated stress at high doses - Generally biocompatible - Iron metabolism considerations
NIR-II Polymer Nanoparticles (Optical probes)	- 20–40 nm - Polymeric - near-neutral charge	- Rapid but sustained peritoneal residence when IP - Favourable plasma PK	- High S/N in orthotopic tumours - Lymphatic drainage mapping - Minimal off-target uptake, due to size & neutrality	- Phototoxicity low - Minimal systemic burden - Stable in ascitic fluid - Low complement activation
GSH-Responsive Pt (IV) Hybrid Nanoparticles	- 100 nm - Responsive shell - Mild negative charge	- Platinum released rapidly only in high GSH - Reduced systemic platinum exposure	- Tumour-localised activation - Low kidney accumulation - Improved uptake with ultrasound-enhanced delivery	- Decreased Nephrotoxicity vs. cisplatin - Good tolerability - Redox trigger ensures minimal off-target toxicity
Hypoxia-Activated Liposomes (CPD100Li)	- 100 nm - Sphingomyelin–cholesterol bilayer	- Hypoxia led to high cytotoxic activation - PK favourable with IP/IV	- Preferential activation in hypoxic peritoneal nodules - Limited liver/kidney penetration - Stable circulation profile	- Good tolerability - Selective toxicity reduces systemic adverse events
Large Anionic TAM-Targeting Liposomes (Resiquimod)	- More than 200 nm - Strongly anionic	- Local TAM uptake dominates PK - Short systemic exposure	- Enriched in peritoneal TAMs - Minimal epithelial tumour uptake - Low systemic distribution	- Transient cytokine induction expected (TLR7/8 agonist) - Combination with PD-1 blockade improves safety–efficacy balance
Zwitterionic or Stealth Nanogels (UCST PTX-gels)	- 60–120 nm - UCST responsive - Zwitterionic	- Long circulation - Low protein adsorption - Heat-triggered release enhances local PK	- Low RES clearance - Minimal corona formation in ascites - Stable peritoneal retention	- Excellent antifouling - Low immunogenicity - Thermal activation avoids high systemic doses
Cell Membrane Coated Nanoparticles (RBC-, leukocyte-, and cancer-derived)	- 100–180 nm - Natural membrane cloaking	- Extended circulation compared to uncoated cores - Reduced opsonisation	- Homotypic targeting enhances tumour tropism - Decreased liver sequestration - Improved ascites navigation	- Potential immunogenicity depending on membrane source - Batch variability - Excellent immune evasion properties

**Table 7:** Clinical translation of ovarian cancer nanomedicines: summary of platforms, trial phases, and reported outcomes.

Nanomedicine Platform/Trial	Phase and Clinical Setting	Mechanism/Technology	Key Clinical Outcomes	Regulatory/Translational Notes
Pegylated Liposomal Doxorubicin (PLD, Doxil or Caelyx)	Phase III: First-Line and Relapsed Ovarian Cancer	PEGylated liposome encapsulating doxorubicin	MITO-2: PLD–carboplatin non-inferior to paclitaxel–carboplatin - Less neurotoxicity and alopecia - More haematologic AEs	- Benchmarked standard for liposomal PK improvement - Long-approved nanomedicine in ovarian cancer
PLD and Trabectedin	Phase III: Platinum-sensitive relapse	Liposomal DOX combined with marine-derived alkylator	- Improved PFS in certain subgroups - Acceptable safety profile vs. PLD alone	- Used clinically - Important example of nanomedicine–chemotherapy combinations
PLD and Bevacizumab	Phase III: Recurrent disease	Liposomal DOX with anti-VEGF antibody	PLD–carboplatin–bevacizumab outperformed gemcitabine–carboplatin–bevacizumab in outcomes and tolerability	Shows synergy between nanomedicine and anti-angiogenic therapy
Nab-Paclitaxel (Albumin-bound paclitaxel)	Phase II or Phase I: - Refractory ovarian cancer - Trials in IP and aerosol delivery	130 nm albumin-nanoparticle taxane	- Notable standalone activity - Favourable PK - Manageable dose-limiting toxicities - Under exploration for IP/PIPAC routes	- Highly scalable manufacturing - Regulatory success model for nanocarriers
EPHARNA (DOPC-siRNA targeting EphA2)	GLP Safety Completed: Preclinical-to-IND Transition	Neutral liposome delivering EphA2-siRNA	- Robust gene silencing and survival benefit in orthotopic models - Safe in primates (no major cytokine elevations)	Among the closest siRNA nanomedicines to clinical entry in ovarian cancer
Hybrid LNP PARP/siRNA platforms, e.g., PLK1-siRNA LNPs, PARP1-targeted LNPs	Preclinical	Lipid nanoparticles delivering RNAi agents	- Marked survival extension in models - Effective in BRCA-wild-type and resistant disease	- Raising interest but lacking human trial data - Regulatory path still early
CRISPR or Cas9 Liposomal Nanomedicine	Preclinical	Liposomal CRISPR plasmid delivery	- Efficient in vivo genome editing - Tumour suppression in xenografts	Demonstrates feasibility but major regulatory hurdles (gene editing)
Radiolabelled Liposomes/ENPs, e.g., ^99^mTc-ENPs, ^188^Re-liposomes	Early Clinical Imaging Studies	SPECT/PET-traceable nanocarriers	- Enabled quantitative PK imaging, tumour uptake mapping, early signs of therapeutic benefit (188Re-liposome)	- Strong theranostic candidates - Regulators receptive due to quantitative imaging
Folate-targeted agents, e.g., vintafolide and etarfolatide imaging	Phase II/III	FRα-targeted drug conjugate and FRα-SPECT tracer	- Companion imaging enabled patient selection - Mixed survival advantages - Halted during Phase III due to stratification issues	Landmark in showing imaging-guided patient enrichment for ovarian nanomedicine
Inhaled/IP nanoparticle taxanes (IP Nab-PTX or aerosolised PTX-NPs)	Phase I	Locoregional nanotherapy	- Better peritoneal retention and lower systemic toxicity - Promising early efficacy in metastatic peritoneal disease	Highlights regulatory discussion around device–drug combination approaches
Iron oxide MRI nanoplatforms (HER2-targeted or cisplatin-tagged)	Translational/First-in-Human Imaging Ready	SPION-based theranostics	Preclinical: - Tumour visualisation and therapy - Early clinical imaging utility under evaluation	Regulatory focus on long-term metal accumulation

## Data Availability

No new data were created or analyzed in this study.
